# Metapopulation structure modulates sexual antagonism

**DOI:** 10.1002/evl3.244

**Published:** 2021-06-30

**Authors:** E. Rodriguez‐Exposito, F. Garcia‐Gonzalez

**Affiliations:** ^1^ Doñana Biological Station (EBD‐CSIC) Isla de la Cartuja Sevilla Spain; ^2^ Current address: Institute of Natural Products and Agrobiology (IPNA‐CSIC) Santa Cruz de Tenerife Spain; ^3^ Centre for Evolutionary Biology, School of Biological Sciences University of Western Australia Crawley Western Australia Australia

**Keywords:** Callosobruchus maculatus, ecological context, evolutionary ecology, experimental evolution, female resistance to male harm, metapopulation structure, population subdivision, sexual conflict, sexual selection, sexually antagonistic coevolution

## Abstract

Despite the far‐reaching evolutionary implications of sexual conflict, the effects of metapopulation structure, when populations are subdivided into several demes connected to some degree by migration, on sexual conflict dynamics are unknown. Here, we used experimental evolution in an insect model system, the seed beetle *Callosobruchus maculatus*, to assess the independent and interacting effects of selection histories associated with mating system (monogamy vs. polygamy) and population subdivision on sexual conflict evolution. We confirm traditional predictions from sexual conflict theory by revealing increased resistance to male harm in females from populations with a history of intense sexual selection (polygamous populations) compared to females from populations with a history of relaxed sexual selection (monogamous populations). However, selection arising from metapopulation structure reversed the classic pattern of sexually antagonistic coevolution and led to reduced resistance in females from polygamous populations. These results underscore that population spatial structure moderates sexual selection and sexual conflict, and more broadly, that the evolution of sexual conflict is contingent on ecological context. The findings also have implications for population dynamics, conservation biology, and biological control.

**Impact Summary**

The reproductive interests of the sexes often differ in sexually reproducing species and this sexual conflict pervades the biology and evolution of the interactions between males and females. Virtually all research on the causes and consequences of sexual antagonism has been conducted using simple population/ecological scenarios. Despite natural populations being frequently subdivided, the role of metapopulation structure on the dynamics of sexual conflict and sexually antagonistic coevolution is unknown. In this study, we address this gap by applying an experimental evolution approach in an insect model system that is characterized by intense sexual conflict. We manipulated selection arising from population spatial structure and connectivity, and also manipulated sexual selection/sexual conflict evolutionary history by enforcing, or not, a monogamous mating system across generations. Subsequently, we investigated sexually antagonistic coevolution trajectories at different points in time under different intensities of sexual interactions. Therefore, we were able to study for the first time the interaction between selection history associated with mating system and selection history related to metapopulation structure. The results of our study support traditional sexual conflict theory by showing that females from populations with a history of intense sexual selection exhibit increased resistance to male harm. However, our study also reveals startling findings: selection arising from metapopulation structure reverses the classic pattern of sexually antagonistic coevolution. Females from subdivided polygamous populations experienced an evolutionary reduction in resistance to male harm. These findings show that metapopulation structure moderates sexual selection and sexual conflict. More broadly, they show that the evolution of conflict between males and females is contingent on ecological context. These results have direct implications for many aspects of sexual selection as well as for population dynamics, conservation biology, and biological control.

The evolutionary interests of males and females over mating and reproduction often differ (Parker 1979; Arnqvist and Rowe 2005). Conflicts concerning traits whose expression is determined by different loci in males and females (inter‐locus sexual conflict) frequently leads to the evolution of traits in one sex (generally males) that are harmful to the opposite sex (Holland and Rice 1999; Pitnick and Garcia‐Gonzalez 2002; Arnqvist and Rowe 2005). Such adaptations in the harming sex may in turn select for counter‐adaptations in the other sex, ultimately leading to sexually antagonistic coevolution (Holland and Rice 1998; Arnqvist and Rowe 2002; Rowe and Day 2006; Dougherty et al. 2017). The evolutionary consequences of sexual conflict and sexually antagonistic coevolution are far from trivial. For instance, male harm to females can result in a tragedy of the commons, whereby strategies shaped by sexual conflict can dramatically reduce population viability (Rankin et al. 2007; Hollis and Houle 2011; Rankin et al. 2011; Berger et al. 2016). This effect on population persistence may be aggravated by the fact that the costs and benefits of sexual conflict are not carried by the same individuals (Kokko and Brooks 2003). Ultimately, sexual conflict may thus lead to population extinction (Le Galliard et al. 2005), but this is just one of the evolutionary implications of sexual conflict, as evidence gathered over the last two decades has also shown that sexual conflict has consequences for the evolution of sexual dimorphism, genomic organization, diversification and speciation (Gavrilets 2000; Arnqvist and Rowe 2005; Rankin et al. 2011; Gavrilets 2014; Sayadi et al. 2019).

Most research on sexual conflict to date, however, has been conducted in uniform environments. It is becoming increasingly evident that the ecological context needs to be considered when assessing the consequences of sexual antagonism (Arbuthnott et al. 2014; Perry et al. 2017; De Lisle et al. 2018; Perry and Rowe 2018; García‐Roa et al. 2019; García‐Roa et al. 2020). In addition, recent research on the effects of dispersal and kin selection on sexual conflict has hinted at the importance of moving beyond studying simple population structures when assessing the evolution of sexual antagonism (Eldakar et al. 2009; Wild et al. 2011; McDonald et al. 2013; Carazo et al. 2014; Pizzari et al. 2015; Faria et al. 2020; Lymbery et al. 2020; Rodrigues et al. 2021). Nevertheless, the role of population subdivision and demes's connectivity on the dynamics of sexual conflict and sexually antagonistic coevolution is unknown. This is surprising since the spatial structuring of populations has been the focus on intense research over the past decades. Natural populations are frequently subdivided into demes of various sizes that are interconnected by migration, and there is now overwhelming evidence that metapopulation structure has far‐reaching evolutionary and ecological consequences (Levin 1974; Hanski 1999; Hanski et al. 2011).

Clear predictions regarding how metapopulation structure may shape sexual conflict dynamics are mostly absent. On the one hand, metapopulation structure may favor the fixation of polyandrous behavior under some conditions, for instance, when temporal or permanent male infertility is pervasive in the population (Garcia‐Gonzalez 2004; Yasui and Garcia‐Gonzalez 2016). Variation in female mating frequency may modulate the total opportunity for sexual selection (Evans and Garcia‐Gonzalez 2016), and an increase in polyandry levels is associated with weaker precopulatory but stronger postcopulatory selection on males (Collet et al. 2012; Morimoto et al. 2019). If rates of female multiple mating are higher in subdivided populations compared to panmictic populations this would then imply that post‐copulatory sexual selection could be more intense in spatially structured populations. Following this logic, sexual conflict could be expected to be more intense in metapopulations, at least in systems where sexual conflict stems to a large extent from adaptations to postcopulatory sexual selection, as is the case in many study models (Wigby and Chapman 2005; Hotzy and Arnqvist 2009; Cayetano et al. 2011; Hotzy et al. 2012; Smith et al. 2017).

On the other hand, sexual conflict theory predicts more intense sexual conflict in larger populations (Gavrilets 2000; Martin and Hosken 2003; Gay et al. 2009) and in fact this seems to be the case (Gay et al. 2010). Indeed, reduced genetic drift and higher levels of genetic variability in large, dense populations, along with higher probabilities for individuals to interact with other individuals (mates and competitors) may fuel sexually antagonistic selection. If so, sexual conflict and the evolutionary chases between the two sexes could be slowed down or impeded at the metapopulation level. Furthermore, subpopulations within a metapopulation can act as refuges for different genotypes or strategies that would otherwise be selected against in non‐spatially structured large populations, for example, coexistence of predators and prey, coexistence of different mating system strategists, and so on (Huffaker et al. 1963; Hassell et al. 1991; Tilman 1994; Holyoak and Lawler 1996; Bonsall et al. 2002; Yasui and Garcia‐Gonzalez 2016). In other words, metapopulation theory predicts that less competitive genotypes can persist for longer under spatial structuring. Applied to sexual conflict scenario, the prediction would be that less resistant females or less competitive males could persist for longer in small subpopulations, implying less intense sexually antagonistic selection in metapopulations.

Here we use experimental evolution in an insect model system exhibiting marked sexual conflict, the seed beetle *Callosobruchus maculatus* (Hotzy and Arnqvist 2009; Gay et al. 2010; Sayadi et al. 2019), to assess for the first time the independent and interacting effects of selection associated with mating system and metapopulation structure on the evolution of male harm and female resistance. We relaxed sexual selection and sexual conflict in eight selection lines by enforcing monogamy, while keeping another eight populations under polygamy (intense sexual selection and conflict). A critical novelty was imposed in the experimental evolution protocol: half the selection lines were maintained under conditions of population subdivision and controlled migration among demes, whereas the remaining populations were kept undivided (Fig. 1). Our design is a simplification of the real world, but it retains key metapopulation features (population subdivision and population connectivity). For simplicity, we use the term metapopulation structure throughout the text to refer to the selection treatment generating subdivided populations connected through migration, but we acknowledge that metapopulations are characterized by more than just fragmentation and limited gene flow (e.g., local stochastic extinction and re‐colonisations, variation in deme size, etc.).

**Figure 1:**
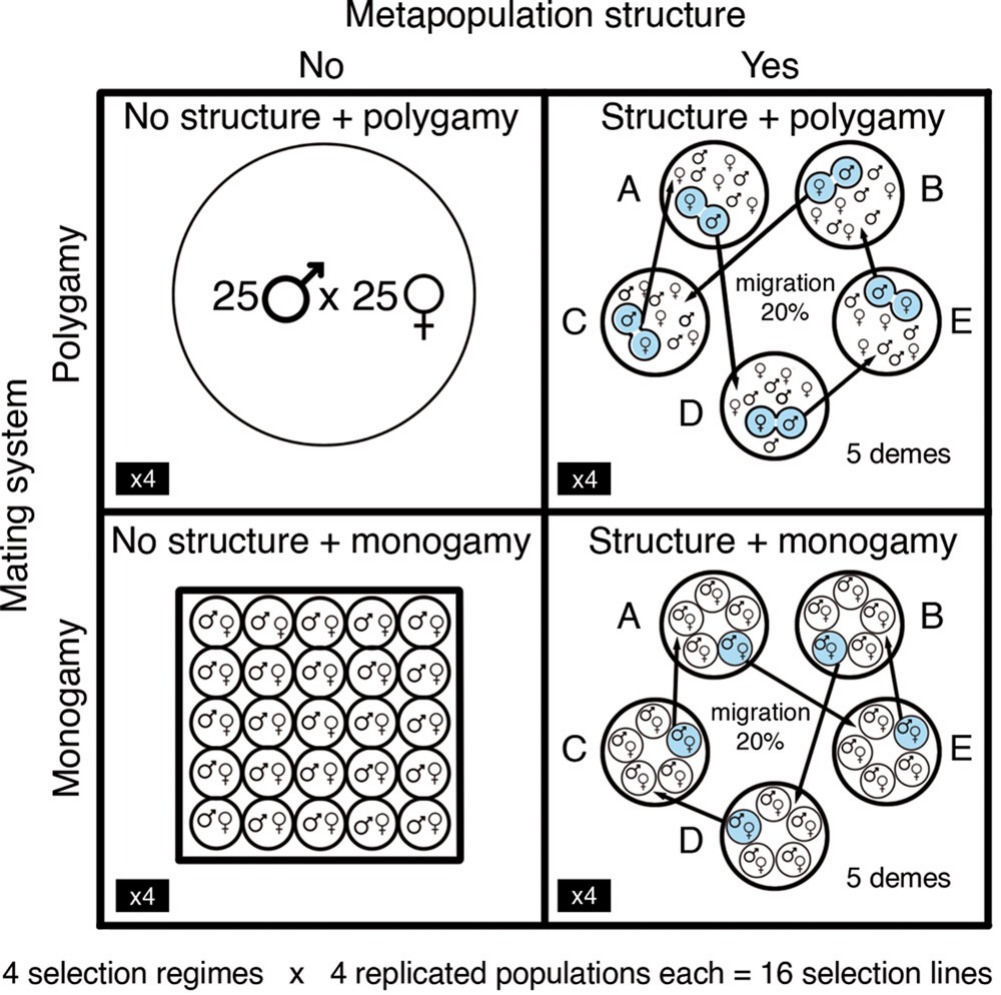
Outline of the experimental evolution protocol. Mating system variation (monogamy vs. polygamy) and variation in metapopulation structure (no vs. yes) were combined to generate four different selection regimes. A single selection line is depicted within each combination of the selection regime treatments for simplicity, although there were four lines per each mating system × metapopulation structure treatment combination (16 selection lines in total). In those cases in which there was metapopulation structure, the selection line was subdivided into five different demes (subpopulations). To allow gene flow, each generation one randomly chosen individual from each sex and subpopulation (highlighted in blue) was transferred to a different subpopulation (determined at random), such that each subpopulation received only one male‐female migrant pair from another deme. The direction of the arrows connecting the subpopulations in the figure is a random depiction of a hypothetical 20% migration scenario

The evolution of male harm and female resistance was inferred through different assays imposing varying levels of male harassment and opportunities for conflict. Results suggest that evolution under metapopulation structure ameliorates the detrimental consequences of sexual conflict for female fitness.

## Methods

### EXPERIMENTAL EVOLUTION PROTOCOL

We used the seed beetle *Callosobruchus maculatus* as a model system. We sourced the beetles from an outbred population (South Indian population) that we established in our laboratory with over 450 individuals and that we keep with large population sizes (in excess of 300 individuals) and non‐overlapping generations (see the Appendix for further details). Previous studies have shown that the South Indian population presents ample phenotypic and genetic variance (Fox et al. 2004; Bilde et al. 2008; Berg and Maklakov 2012; Rodriguez‐Exposito 2018; Zajitschek et al. 2018; Canal et al. 2021). We set up an experimental evolution protocol consisting in the combination of two selection treatments with two levels each: metapopulation structure (no vs. yes) and mating system (monogamy vs. polygamy; i.e., relaxed or intense sexual selection/conflict, respectively; see below and Fig. 1). Briefly, eight populations (henceforth metapopulation lines) derived from the stock population were each subdivided into five subpopulations, while another eight populations derived from the same source population were kept as controls (i.e., undivided). Individuals from the source population were randomized among these groups, keeping an equal sex ratio, and all the populations were originated and maintained with 50 breeders each (25 females and 25 males). Importantly, half of the lines in each level of the population structure treatment were kept under enforced monogamy and the other half under polygamy, where sexual interactions and matings occurred in an unrestricted way among all the individuals in each population/subpopulation. Thus, the selection experiment consisted of a 2×2 design (sexual selection x metapopulation structure experimental evolution treatments), with 16 lines (see Fig. 1): four lines under polygamy (intense sexual selection) and absence of metapopulation structure: non‐structure and polygamy lines, henceforth *NSPoly* lines; four lines kept under metapopulation structure and polygamy, henceforth *SPoly* lines; four lines with a selection history of absence of metapopulation structure and monogamy (relaxed sexual selection and conflict), henceforth *NSMono* lines; four lines under metapopulation structure and monogamy: henceforth *SMono* lines. While there is some scope for choice and harm phenomena to affect females' resource provisioning to eggs in the monogamous lines, enforced monogamy effectively relaxes sexual selection and ensuing sexual conflict to a large degree, because in these lines precopulatory or postcopulatory paternity biases due to female choice or male‐male competition is absent. In addition, the mating system treatment can alter natural selection on females via biased allocation of male harm (Long et al. 2012), and monogamy can also work to reduce intralocus sexual conflict (Hollis et al. 2014). The assays described below were carried out at generations 12, 30, 32, 43, and 47 of the selection experiment.

A critical aspect of metapopulation structure is the presence of gene flow among demes (Hanski 1998; Hanski 1999; Hanski and Gaggiotti 2004). In each generation, one randomly chosen individual from each sex and subpopulation (i.e., one male and one female per each of the 5 subpopulations within each subdivided population) were forced to migrate (i.e., were relocated) to a different subpopulation within each metapopulation line (Fig. 1). This imposed 20% migration rate was carried out upon adult emergence using virgin individuals.

Using virgin individuals, we allowed sexual interactions and ensuing egg laying by females for 2 days across all lines (Day 1 and Day 2 of the experimental cycle). The number of beans per female was standardized (see Appendix) to ensure ad libitum oviposition substrate so as to make sure that larval competition was mostly absent (Fox and Messina 2018). On Day 3, the breeding individuals were removed from the containers hosting the animals. On Day 10, we randomly selected 150 inoculated beans from each line. The great majority of the inoculated beans across all treatments had only one egg per bean, indicative of a lack of competition among females for oviposition substrate. Each of the randomly selected inoculated beans (n = 2400 per generation across the 16 lines) was isolated into an eppendorf tube with pinholes in the cap to allow airflow and kept until adult emergence (starting typically on Day 21). This protocol ensured virginity of the individuals to be used as breeders for the next generation. Virgin adults were collected randomly among those emerged between Day 21 and Day 24 of the cycle so as to avoid inadvertent selection on development time (Maklakov et al. 2010; Cayetano et al. 2011). Typically nearly all individuals emerged within these days. At this point the cycle started again, setting up 1–4 day‐old individuals as breeders for the next generation (Day 1 of the experimental cycle). Further details about the propagation of lines can be found in the Appendix.

Apart from the measures of baseline longevity, all the assays were run on the individuals after one or two generations of common garden breeding. To this end, we duplicated the number of inoculated beans collected from each line (i.e., 4800 beans in total). We then established a set of 16 replicated common garden lines all under *NSPoly* conditions. In this way divergence in the traits assayed could be attributed to direct genetic effects rather than to maternal/paternal or other environmental effects arising from the type of breeding (Simmons and Garcia‐Gonzalez 2008; Garland and Rose 2009; Kawecki et al. 2012).

### EFFECTIVE POPULATION SIZES (*N_e_*)

In each generation, each selection line was seeded with 25 females and 25 males. Effective population sizes (*N_e_*) may, however, vary across selection regimes. We estimated effective population sizes using different published methods, and also implemented Monte Carlo simulations adjusted to each selection regime to mimic the precise extraction protocol to obtain the breeding individuals that we followed in our selection experiment. We also implemented in our calculations scenarios where infertile matings were considered (see Appendix for full details). None of the different methods to estimate *N_e_* returned significant differences in effective population sizes among the different selection conditions, and the estimates (the majority of them ranging between 41–51), are not much lower than the census population size (see Tables A2‐A4 in the Appendix). Overall, population subdivision yielded slightly higher *N_e_* than conditions with no spatial structure (see also Walsh and Lynch 2018). In addition, the relaxation of sexual selection (enforced monogamy) resulted in slightly higher *N_e_* compared to polygamous conditions. Besides the broader potential implications of these findings (e.g., population spatial structure increases to some extent *Ne*, and sexual selection decreases *Ne*), the slight variation between selection regimes are unlikely to lead to differences in inbreeding or drift in our lines, and the empirical evidence thus far indicates so. First, there are no differences in the prevalence of infertility among selection regimes (Rodriguez‐Exposito 2018). Second, there are no differences in male or female baseline longevity among selection regimes (this study). Third, there are no differences in net reproductive rates among selection regimes either (Rodriguez‐Exposito 2018). In sum, differences among treatments in the responses investigated in this study are attributable to selection.

### TESTER INDIVIDUALS

The evolution of male harm and female resistance was assayed on focal animals mated to tester individuals that were sourced from outside the selection experiment. In this way, confounding effects due to within‐line male‐by‐female coevolution were controlled for. To standardize the background upon which traits in experimentally evolved beetles were measured, tester individuals were obtained from near‐isogenic lines. This degree of experimental control is especially important when assaying traits that are determined by sexual interactions because it minimizes sampling variance arising from the random sampling of mates differing in their effects on the traits measured in the focal individuals (see for rationale and application Garcia‐Gonzalez and Evans 2011; Garcia‐Gonzalez and Dowling 2015; Travers et al. 2015). The Appendix provides specific details about the generation of tester individuals.

### EVOLUTION OF MALE INDUCED HARM AND FEMALE RESISTANCE

The evolution of male harm and female resistance was investigated by assessing the effects of mating interactions on female fitness, namely female longevity and female lifetime reproductive success (LRS). The evolution of male harm was inferred according to the effects of focal males on tester female fitness, while the effects of tester males on focal female fitness informed on the evolution of female resistance. A series of assays and experiments were run:

#### No sexual conflict assay: Baseline longevity

Differences among the different replicated populations in female longevity responses following sexual interactions can be due to intrinsic survival differences, rather than to differences attributable to the ability of females to resist harm. To take into account this possibility we measured baseline longevity in the selection lines (generation 43). We monitored lifespan in virgin focal females and males when they were in isolation, excluding therefore the influence of mate‐driven factors affecting longevity. Individuals from the different selection lines were collected from infested beans that were individually isolated in 1.5 ml Eppendorf tubes with pin holes for ventilation. These tubes were monitored on a daily basis for adult emergence and subsequent adult longevity. Longevity was measured in a total of 944 individuals distributed in approximately 30 individuals from each sex per line (n = 472 males and 472 females; see Table A1). Elytron length, a proxy for body size, was measured in all individuals (see Appendix).

#### Low levels of sexual conflict assay: Female LRS and longevity after single mating

These assays were carried out at generation 32 of the selection experiment using individuals sourced after two generations of common garden. The assays focused on measuring LRS and longevity after a single mating in 2–3 days‐old females that were either focal females mated to tester males (thereby providing a measure of female resistance to male‐induced harm), or tester females mated to focal males (thereby providing a measure of male‐induced harm). For each of these two assays, 10 focal virgin individuals were randomly selected per each of the 16 replicated lines (total number of focal individuals across the two sexes screened = 320; Table A1). Each focal individual was paired with a single tester individual of the opposite sex in a small glass container (10mm diameter × 40 mm high). Each couple was allowed 15 min to initiate mating. After mating the male was removed and the female was transferred to a small plastic container (30 ml) with ad libitum beans to allow oviposition. Females remained in these containers until their death. Longevity data was recorded on a daily basis and the containers with the newly emerged adult offspring were frozen 29 days after their establishment, allowing enough time for the emergence of the majority if not all offspring produced by females; a vast majority of offspring from singly mated females is produced in the first 1–3 days after mating (Zajitschek et al. 2018). Adult offspring production was later counted. Body size was measured in all focal and tester males and females. Figure A1 provides an outline for the experiment.

#### Medium levels of sexual conflict assay: Female longevity under variable female mating rates

In an additional experiment, we assessed female resistance by looking at female longevity when controlling for variable female mating rates. These assays were run at generations 12 and 30, after one generation of common garden breeding. Seven females per each of the 16 selection lines at generation 12 (n = 112), and 10 females per replicated line at generation 30 (n = 160), were tested (final sample size for the analyses across the two generations was 262 females; see Table A1). All focal females were 1–3 days old. Each focal female was allowed to mate with a single tester virgin male (see details in the Appendix) for 15 minutes once daily for 12 (generation 12), or 10 (generation 30) days. All mating opportunities were given in the mornings in clean glass vials (38 mm high, 10 mm diameter). After 15 min the male was removed and the female was transferred to a 1.5 ml Eppendorf tube until the next mating opportunity (with a different male) 24 h later. Females were monitored daily for survival. Females did not have access to oviposition substrate at any time during the assays or afterward. Under these conditions, longevity is extended compared to situations where females can lay eggs (see below and Messina and Fry 2003). No female died before completing the mating opportunities period. Body size of experimental females was measured (Appendix). Figure A2 provides an outline of these assays.

#### Extreme levels of sexual conflict assay: Female LRS and longevity under continuous exposure to intense male harassment

Male harm to females and female resistance to male harm following lifelong sexual cohabitation was measured in beetles after 47 generations followed by one generation of common garden. Male harm was estimated in assays in which we measured LRS and female longevity of standardized tester females housed with focal males. The evolution of the female's ability to resist male harm was estimated in assays where LRS and longevity were assessed in focal females when they were housed with standardized tester males (Fig. A3). Each female (approx. 2 days‐old) was housed with three males (1‐4 days old) in a small plastic container (30 ml approx.) with approx. 85 beans for the first 24 hours (day 1). On the second day, the individuals were transferred to a second container with the same amount of beans, where they remained for a week. Individuals were then transferred to a third container with a similar amount of beans, where they remained until female death. This protocol ensured that females had beans *ad libitum* to lay eggs. Males that died before the female were replaced with new males to keep the ratio of males to females in all containers constant. The containers were kept for 29 days after the removal of the experimental individuals, or after female death, to ensure that all offspring completed development and emerged as adults. At that moment the containers were frozen at −20°C for later counting of LRS. The body size of all females was measured. Male‐induced harm was measured in 10 replicate units per selection line, and female resistance in additional 10 replicates per selection line (n = 320 replicated units across both tests; Table A1).

### STATISTICAL ANALYSES

Linear Mixed Models (LMMs) were fitted using the function *lmer* in the package *lme4* (Bates et al. 2015). In the analysis of fitness traits after single mating or continuous male‐female cohabitation we had data on the evolution of male harm (fitness of tester females mated to focal males), and data on the evolution of female resistance (fitness of focal females mated to tester males). The response variables in this group of assays were female longevity and LRS (squared). We also measured baseline longevity data in virgin individuals. As predictors, the models included the mating system experimental evolution treatment (two levels), the metapopulation structure experimental evolution treatment (two levels), and the interaction of these two factors, as fixed effects. Body size was included as a covariate in all the analyses, and in the analyses of responses in male harm and female resistance we also included the second order interactions between male or female body size and the treatments because male body size might be associated with male harm and female body size with female resistance (see results, Appendix, and Pitnick and Garcia‐Gonzalez 2002), and the influence of body size on harm/resistance could be expected to differ according to treatment. To account for reproduction‐survival relationships (Williams 1966; Messina and Slade 1999; Roff 2002; Messina and Fry 2003; Canal et al. 2021), female longevity was included as a covariate in the analyses of LRS, while LRS was included in the models where female longevity was the response variable. While the experimental procedures ensured minimal variation in female age when entering the assays (see Appendix), this variable was also included, as a control covariate in the models.

In the assays with variable female mating frequency, only female longevity was measured, as in these assays females were not allowed to lay eggs. Predictors in these models included the two experimental evolution treatments, female body size, and the second order interactions involving these three predictors. Age, generation, and female mating frequency were included as control covariates. Covariates were mean‐centered in all analyses (Schielzeth 2010).

Line ID was included as a random effect in the models. We fitted by‐line ID random intercept and random slopes models to avoid inflation of type I error (Schielzeth and Forstmeier 2009; Barr et al. 2013; Bates et al. 2015; Arnqvist 2020). The correlations between intercept and slopes were also included in the models, excepting when there were issues of convergence in complex models (see Barr et al. 2013 and extended methods in the Appendix). For covariates that were included in the model as control predictors random slopes were not modeled (Barr et al. 2013 and see Appendix).

Significance of the fixed effects in LMMs was calculated with Type II (Type III when interactions are significant) Wald Chi‐square tests, on maximum likelihood models, while parameter estimates were calculated using Restricted Maximum Likelihood (Zuur et al. 2009). We report mean ± standard error values throughout. Complete details on statistical analyses can be found in the Appendix. Final sample sizes are in Table A1, and Table A5 shows the means for the response variables for the different selection regimes across experiments.

## Results and Discussion

### NO SEXUAL CONFLICT ASSAY

The different selection regimes did not lead to divergence in the longevity of virgin females, which was only influenced by female body size (Appendix, Table A6, Fig. A4).

### LOW LEVELS OF SEXUAL CONFLICT ASSAY

The evolution of male harm under low levels of sexual conflict was assessed in experimental assays in which females were mated singly. In such tests, female body size was the only variable significantly predicting female longevity. The LRS of females was only influenced (positively) by the body size of their mates (Table 1), suggesting the existence of direct benefits from mating associated with the receipt of (larger) ejaculates (Savalli and Fox 1999; Zajitschek et al. 2018). These findings thus support the view that mating is both beneficial and costly (see below) for females, and that the outcome of sexual interactions is the result of a complex balance.

**Table 1:** Evolution of male harm (effects of focal males on tester females) assessed after single copulations: Linear mixed models (LMMs) on the effects of focal males on the longevity and LRS of tester females with whom they mated singly. Monogamy is the reference level for the mating system treatment, and no population subdivision is the reference level for the metapopulation structure treatment. Due to convergence issues with the maximal models, the correlations between random intercept and random slopes were removed, to simplify the random effects structure of the models (see text). P‐values were calculated using type II sums of squares on maximum likelihood models, while parameter estimates were calculated using REML models. P‐values in bold are significant at <0.05

**Fixed predictors**	*β*	*Type II Wald χ2*	*Wald test df*	*P‐value*
**Longevity**				
Intercept	8.11			
Mating system treatment [Poly.]	0.18	0.08	1	0.7780
Metapopulation structure treatment [Yes]	0.25	0.73	1	0.3928
Female body size	2.78	11.57	1	**<0.001**
Male body size	−0.21	0.00	1	0.9969
LRS	−0.01	2.37	1	0.1233
Mating system * Metapop. structure	−0.16	0.02	1	0.8976
Mating system * Female body size	2.66	1.61	1	0.2045
Mating system * Male body size	−1.13	0.15	1	0.6952
Mating system * LRS	0.01	0.23	1	0.6298
Metapop. structure * Female body size	−1.37	0.38	1	0.5389
Metapop. structure * Male body size	1.57	0.32	1	0.5689
Metapop. structure * LRS	0.00	0.11	1	0.7362
**LRS**				
Intercept	3205.43			
Mating system treatment [Poly.]	−146.83	0.10	1	0.751
Metapopulation structure treatment [Yes]	−223.80	0.02	1	0.896
Female body size	965.45	2.24	1	0.134
Male body size	3970.13	6.56	1	**0.010**
Longevity	−151.92	1.58	1	0.208
Mating system * Metapop. structure	451.58	0.78	1	0.376
Mating system * female body size	2351.27	1.08	1	0.298
Mating system * male body size	−2478.79	0.33	1	0.567
Mating system * LRS	11.80	0.03	1	0.852
Metapop. structure * female body size	−369.27	0.00	1	0.953
Metapop. structure * male body size	2982.37	1.37	1	0.241
Metapop. structure * LRS	21.61	0.01	1	0.921

**Figure 2:**
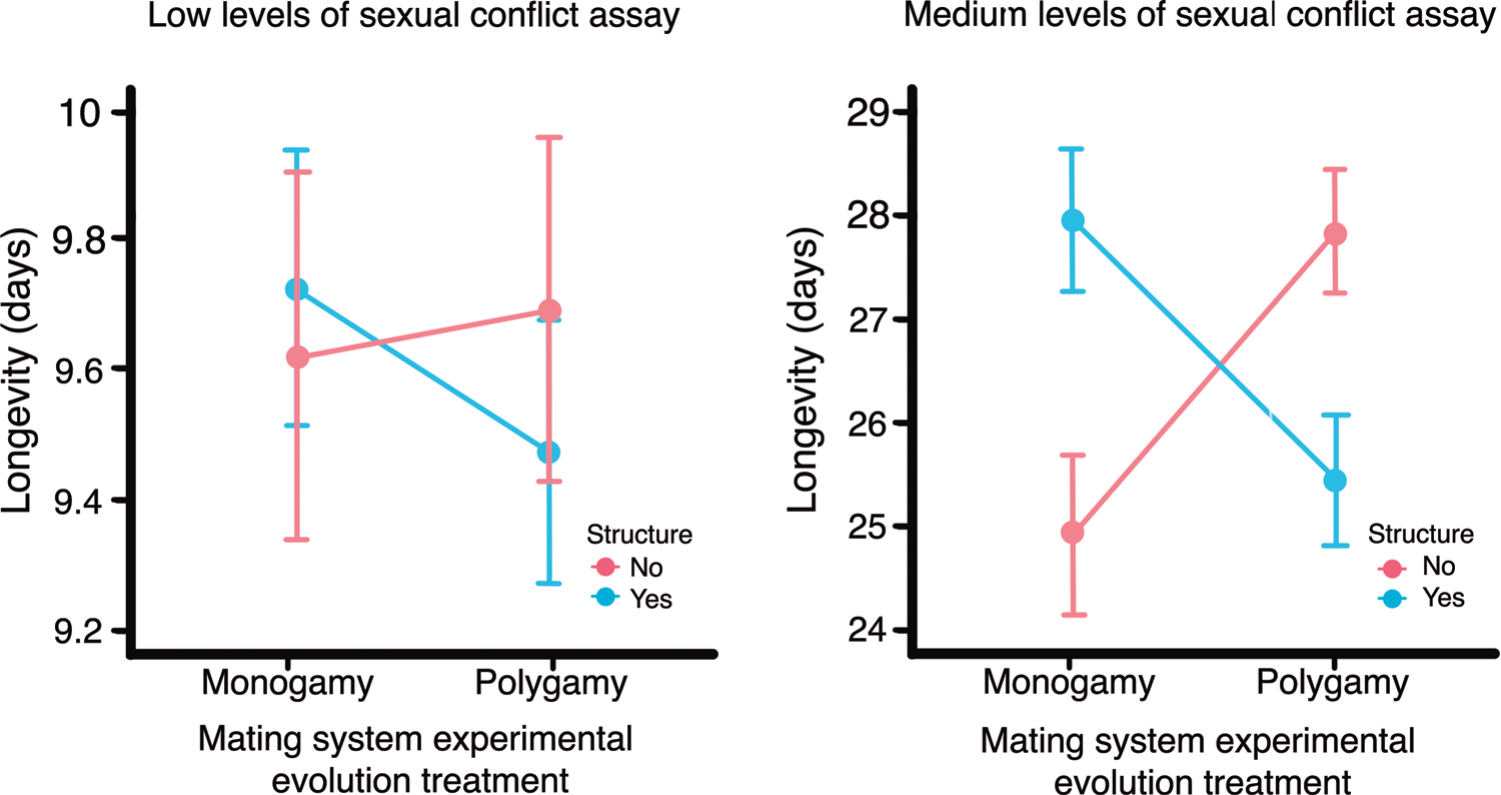
Interaction between mating system and presence/absence of metapopulation structure in the evolution of female resistance in assays with low (left) or medium (right) levels of sexual conflict. Means and SEs (bars) are depicted. Note that the differences in absolute longevity across assays respond to the differences in whether females had access to oviposition substrate (see text): meaningful comparisons in female longevity across selection regimes are those within assays

In the evolution of female resistance test, both female longevity and LRS were positively and significantly influenced by female body size (Table 2). The interaction between the mating system and the metapopulation structure experimental evolution treatments on female longevity was not significant. However, the point estimates are in the same direction as the statistically significant interaction under medium levels of sexual conflict (Fig. 2 and see below). The uncertainty in those estimates under low conflict is, nevertheless, substantial. There was an effect of metapopulation structure increasing LRS (χ^2^_1_ = 5.93, p = 0.015; Table 2, Fig. A5). Results also showed the existence of a cost of reproduction in the form of a trade‐off between LRS and longevity, as expected in this species (Messina and Slade 1999; Messina and Fry 2003; Ronn et al. 2006; Fig. A6).

**Table 2:** Evolution of female resistance (fitness of focal females when mated to standardized males) assessed after single copulations: Linear mixed models (LMMs) on the effects of tester males on the longevity and LRS of focal females after single mating. Monogamy is the reference level for the mating system treatment, and no population subdivision is the reference level for the metapopulation structure treatment. Due to convergence issues with the maximal models the correlations between random intercept and random slopes were removed in the LRS model (see text). *P*‐values were calculated using type II sums of squares on maximum likelihood models, while parameter estimates were calculated using REML models. *P*‐values in bold are significant at <0.05

**Fixed predictors**	*β*	Type II Wald *χ2*	Wald test *df*	*P*‐value
**Longevity**				
Intercept	9.17			
Mating system treatment [Poly.]	0.43	2.95	1	0.086
Metapopulation structure treatment [Yes]	0.40	3.39	1	0.065
Female body size	5.49	18.41	1	**<0.001**
Male body size	−1.17	0.09	1	0.765
LRS	−0.04	14.31	1	**<0.001**
Mating system * Metapop. structure	0.00	0.02	1	0.891
Mating system * Female body size	−0.25	0.03	1	0.865
Mating system * Male body size	0.75	0.06	1	0.810
Mating system * LRS	−0.01	0.44	1	0.509
Metapop. structure * Female body size	−0.14	0.03	1	0.863
Metapop. structure * Male body size	3.07	1.57	1	0.210
Metapop. structure * LRS	0.01	0.54	1	0.464
**LRS**				
Intercept	4441.01			
Mating system treatment [Poly.]	−197.31	0.49	1	0.482
Metapopulation structure treatment [Yes]	251.45	5.93	1	**0.015**
Female body size	5660.15	4.86	1	**0.027**
Male body size	−2366.07	0.05	1	0.821
Longevity	−370.53	24.30	1	**<0.001**
Mating system * Metapop. structure	1040.18	2.31	1	0.128
Mating system * Female body size	−3573.76	0.55	1	0.458
Mating system * Male body size	3442.93	0.69	1	0.406
Mating system * Longevity	−195.06	0.81	1	0.367
Metapop. structure * Female body size	1999.97	0.25	1	0.620
Metapop. structure * Male body size	517.87	0.03	1	0.873
Metapop. structure * Longevity	−92.33	0.14	1	0.713

In summary, there seemed to be little scope for the detection of male harm or female resistance in singly‐mated individuals. This is not surprising, as associations between adaptations to sexual conflict and large fitness advantages (for males) or costs (for females) are unlikely when the consequences of single mating are inspected, given that at least one mating is needed for reproduction (Martin et al. 2004).

### MEDIUM LEVELS OF SEXUAL CONFLICT ASSAY

We found a significant interaction between the mating system and the metapopulation structure evolutionary treatments on female longevity (χ^2^_1_ = 7.77, p = 0.005; Fig. 2, Table 3). In undivided populations, the results support predictions from sexual conflict theory. Females from polygamous populations, who are expected to evolve higher resistance to the damaging effects of harassment and male adaptations that are pushed in polygamous populations, lived longer than monogamous females when mating with standardized males (Fig. 2; and see Gay et al. 2010). However, the pattern was reversed in females from subdivided populations. The interaction was consistent across generations (Fig. A7). Female longevity was positively influenced by female body size, regardless of selection history (Table 3, Fig. A8).

**Table 3:** Evolution of female resistance (longevity of focal females when mated to standardized males) assessed under variable mating rates: Linear mixed models (LMMs) on the longevity of focal females mated to tester males. Monogamy is the reference level for the mating system treatment, no population subdivision is the reference level for the metapopulation structure treatment, and generation 12 is the reference level for generation. Female age when entering the assays, number of matings, and generation are control predictors. *P*‐values were calculated using type III sums of squares on maximum likelihood models, while parameter estimates were calculated using REML models. *P*‐values in bold are significant at <0.05

**Fixed predictors**	*Β*	Type III Wald *χ*2	Wald test *df*	*P*‐value
Intercept	24.15			
Mating system treatment [Poly.]	2.01	4.19	1	**0.041**
Metapopulation structure treatment [Yes]	2.17	4.82	1	**0.028**
Generation	2.21	6.13	1	**0.013**
Female age when entering assay	−0.07	0.00	1	0.994
Number of matings	−0.38	0.92	1	0.336
Female body size	30.14	17.38	1	**<0.001**
Mating system * Metapop. structure	−3.90	7.77	1	**0.005**
Mating system * female body size	−11.90	1.32	1	0.250
Metapop. structure * female body size	−0.58	0.02	1	0.885

The reversal of female resistance patterns cannot be explained by differences in intrinsic longevity (see above). There are no sizeable differences in effective population sizes, calculated using different methods including Monte Carlo simulations, among the different selection conditions (see Methods). This, together with additional lines of evidence including that i) male and female infertility rates are below 3% in all 16 selection lines (Rodriguez‐Exposito 2018), ii) there are no differences in male or female baseline longevity among the 16 replicated populations, and iii) selection regimes has not led to divergence in net reproductive rates (Rodriguez‐Exposito 2018), indicates that the reversal of female resistance patterns cannot be attributed to inbreeding or genetic drift effects.

### EXTREME LEVELS OF SEXUAL CONFLICT ASSAY

**Figure 3:**
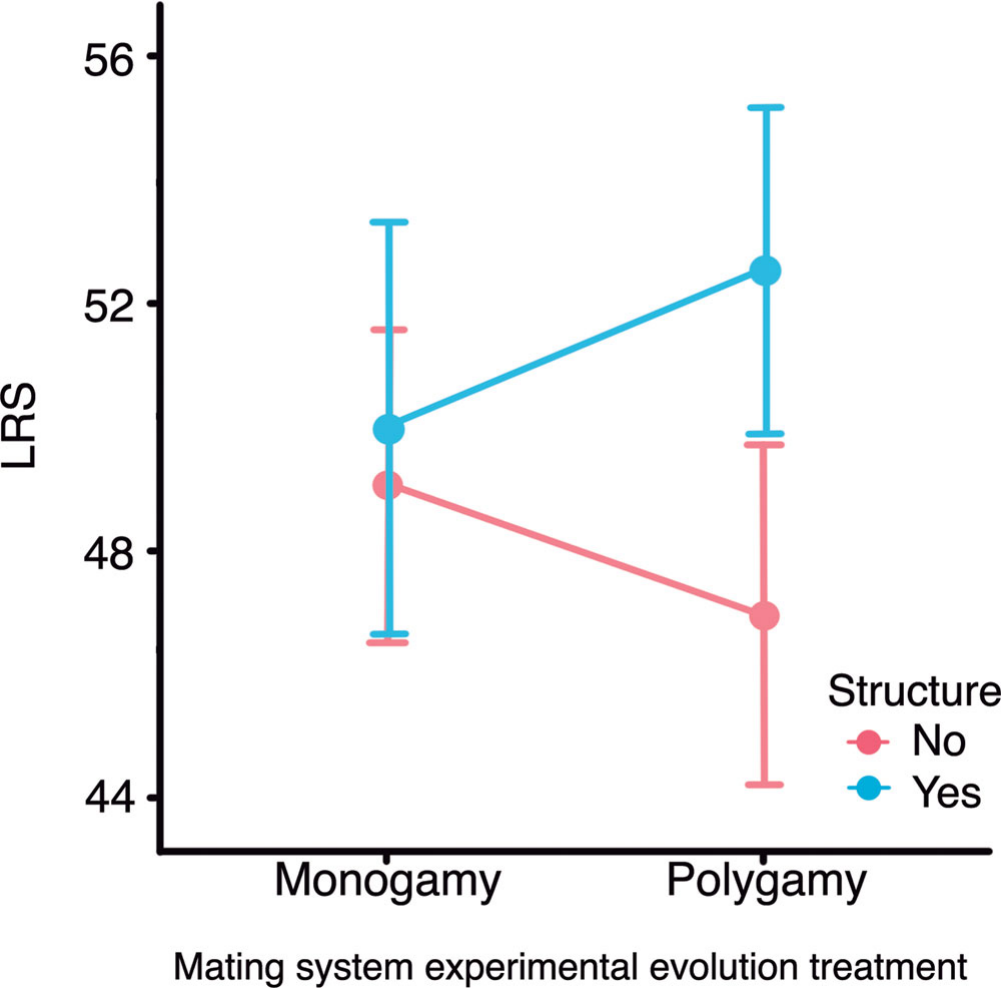
Interaction between mating system and metapopulation structure in the evolution of male effects on tester female's LRS in assays with lifelong continuous male‐biased sexual cohabitation. Means and SEs (bars) are shown

In these tests, each female was exposed to continuous lifelong male harassment and mating attempts from three males. In the tests of the evolution of male harm, there was a significant interaction between the mating system and the metapopulation structure treatments on female LRS, suggesting, again, a reversal of the consequences of sexual conflict. In undivided populations, males from polygamous lines imposed higher harm to tester females (females exhibited lower LRS) than males from monogamous lines, as expected (e.g., see Martin and Hosken 2004). However, this pattern was reversed in subdivided populations (χ^2^_1_ = 4.43, p = 0.035; Table 4, Fig. 3). This pattern mirrors the reversal patterns shown in the other assays for longevity, and it is also reminiscent of findings in one of the few empirical studies on the effects of population size on sexual conflict (Gay et al. 2010). That study documented a relationship between the degree of male‐induced genital damage experienced by females and their LRS that was dependent on whether males had evolved in larger versus small populations, with the cost of genital damage on LRS being only apparent in females mated to males from larger populations. Our results suggest that the female benefit/cost balance derived from sexual selection adaptations in males is relatively more inclined towards benefits in spatially structured populations than in panmictic populations. Additional results from the extreme levels of sexual conflict assays can be found in Table 5 (analysis of female resistance), and in the Appendix, but we are cautious when interpreting the outcomes of our assays under conditions of continuous male‐biased sexual cohabitation. This is so because the housing conditions in these assays (three males per female in a confined space for life) may have led to unrealistic heightened levels of harassment, at the same time that it may have compromised copulations due to high levels of disruptions of mating attempts by competitor males.

**Table 4:** Evolution of male harm (effects of focal males on tester females) assessed under continuous male‐female exposure: Linear mixed models (LMMs) on the effects of focal males on the longevity and LRS of tester females with whom they cohabited during female lifetime. Monogamy is the reference level for the mating system treatment, and no population subdivision is the reference level for the metapopulation structure treatment. Female age when entering the assays is a control predictor. Due to convergence issues with the maximal models, the correlations between random intercept and random slopes were removed, to simplify the random effects structure of the models (see text). *P*‐values were calculated using type II (longevity model) or type III (LRS model) sums of squares on maximum likelihood models, while parameter estimates were calculated using REML models. *P*‐values in bold are significant at <0.05

**Fixed predictors**	*β*	Wald *χ2*	Wald test *df*	*P*‐value
**Longevity**				
Intercept	7.76			
Mating system treatment [Poly.]	0.02	3.34	1	0.068
Metapopulation structure treatment [Yes]	0.69	3.11	1	0.078
Female body size	3.46	13.62	1	**<0.001**
LRS	0.04	23.66	1	**<0.001**
Female age when entering assay	1.12	99.98	1	**<0.001**
Mating system * Metapop. structure	−0.67	3.12	1	0.077
Mating system * Female body size	−1.95	0.85	1	0.356
Mating system * LRS	−0.01	0.99	1	0.320
Metapop. structure * Female body size	2.38	1.20	1	0.273
Metapop. structure * LRS	−0.01	0.71	1	0.399
**LRS**				
Intercept	2927.53			
Mating system treatment [Poly.]	−233.94	0.38	1	0.540
Metapopulation structure treatment [Yes]	−531.21	2.52	1	0.113
Female body size	1604.67	0.42	1	0.516
Longevity	673.77	15.44	1	**<0.001**
Female age when entering assay	−748.73	15.01	1	**<0.001**
Mating system * Metapop. structure	1017.32	4.43	1	**0.035**
Mating system * Female body size	1687.08	0.28	1	0.594
Mating system * Longevity	−160.97	0.82	1	0.367
Metapop. structure * Female body size	1569.93	0.50	1	0.479
Metapop. structure * Longevity	−186.16	1.23	1	0.267

**Table 5:** Evolution of female resistance (fitness of focal females when mated to standardized males) assessed under continuous male‐female exposure: Linear mixed models (LMMs) on the effects of tester males on the longevity and LRS of focal females with whom they cohabited during lifetime. Monogamy is the reference level for the mating system treatment, and no population subdivision is the reference level for the metapopulation structure treatment. Female age when entering the assays is a control predictor. Due to convergence issues with the maximal models, the correlations between random intercept and random slopes were removed in the LRS model (see text). *P*‐values were calculated using type III sums of squares on maximum likelihood models, while parameter estimates were calculated using REML models. *P*‐values in bold are significant at <0.05

**Fixed predictors**	*β*	Type III Wald *χ2*	Wald test *df*	*P*‐value
**Longevity**				
Intercept	7.56			
Mating system treatment [Poly.]	−0.08	0.14	1	0.707
Metapopulation structure treatment [Yes]	−0.03	0.02	1	0.891
Female body size	0.03	0.00	1	0.983
LRS	0.06	37.55	1	**<0.001**
Female age when entering assay	0.92	7.80	1	**0.005**
Mating system * Metapop. structure	0.51	2.83	1	0.093
Mating system * Female body size	−1.90	0.83	1	0.363
Mating system * LRS	−0.04	15.94	1	**<0.001**
Metapop. structure * Female body size	4.95	5.76	1	**0.016**
Metapop. structure * LRS	−0.02	3.99	1	**0.046**
**LRS**				
Intercept	5121.78			
Mating system treatment [Poly.]	221.99	0.29	1	0.588
Metapopulation structure treatment [Yes]	−250.28	0.53	1	0.465
Female body size	6980.48	4.31	1	**0.038**
Longevity	926.77	20.53	1	**<0.001**
Female age when entering assay	−387.54	0.77	1	0.380
Mating system * Metapop. structure	675.48	1.56	1	0.212
Mating system * Female body size	5382.78	2.15	1	0.142
Mating system * Longevity	−640.67	5.79	1	**0.016**
Metapop. structure * Female body size	2969.26	0.79	1	0.373
Metapop. structure * Longevity	−327.12	2.11	1	0.146

### GENERAL CONSIDERATIONS

Sexual conflict theory predicts more intense sexual conflict in larger compared to smaller populations (Gavrilets 2000; Martin and Hosken 2003; Gay et al. 2010). In principle, a similar expectation should apply when comparing undivided and divided populations. For example, in our selection experiment, each individual from a polygamous non‐structured population was able to interact with 25 individuals of the other sex and had to compete with another 24 individuals of the same sex. In structured populations, these numbers are five and four, respectively, for sexually interacting individuals and direct competitors, for each animal. This should impose differences in the intensity of sexual selection and effects due to hard (undivided populations) vs. soft (divided populations) selection (Saccheri and Hanski 2006), ultimately leading to increased sexual conflict and opportunity for sexually antagonistic coevolution in polygamous undivided lines. This is indeed the pattern we observed. These findings support the notion that demes within a metapopulation can act as refuges for different genotypes or strategies that would otherwise be selected against in panmictic populations (Bonsall et al. 2002; Yasui and Garcia‐Gonzalez 2016). In our case, the implication is that less competitive genotypes (i.e., less resistant females, or less harmful males) could persist for longer in metapopulations. In monogamous populations, however, structuring should have no bearings on sexual conflict intensity *per se*, but differences regarding hard (undivided populations) versus soft (subdivided populations) selection may still exist. These differences may underlie the maintenance of high levels of female resistance following the release from sexual selection in monogamous structured populations.

Genetic relatedness among competitors is known to moderate sexual conflict (Carazo et al. 2014; Pizzari et al. 2015; Łukasiewicz et al. 2017; Lymbery and Simmons 2017). If kin selection played a role in our selection experiment, the expectation would be for subdivided polygamous populations to experience less intense conflict than polygamous panmictic populations (but see also Faria et al. 2020), a prediction indistinguishable from the expectation that the intensity of sexual conflict should increase with population size (in terms of the maximum number of interacting individuals). However, amelioration of sexual conflict through kin selection cannot explain the reversal of sexually antagonistic patterns because it would predict no differences among undivided and subdivided monogamous populations (among other things, in monogamous populations individuals do not compete with individuals varying in relatedness, a prerequisite for kin selection to work). Furthermore, even considering only polygamous populations, it is unlikely that the patterns are explained by kin selection resulting from individuals locally competing with individuals that differ in genetic relatedness, since we imposed a 20% migration rate among demes in structured populations every generation. Thus, gene flow would have prevented genetic structuring to a large extent, thus reducing the scope for kin selection as well as differences among lines in inbreeding depression (see above and Appendix). On a different note, we were careful to impose equal and controlled migration rates for both sexes among demes, and thus our results cannot be explained by the effects of sex‐biased dispersal (Eldakar et al. 2009; Faria et al. 2015; Lymbery et al. 2020).

We have provided insights into sexual conflict evolution in subdivided populations that are connected by migration. Beyond confirming that sexually antagonistic arms races ensue in scenarios where sexual conflict is intense, our results reveal unexpected important consequences of population spatial structure on sexual selection and sexual conflict. In addition, they suggest that metapopulation structure modulates relationships (including trade‐offs) between lifespan and other life‐history traits. Broadly, our results underscore the importance of the ecological context in moderating adaptations and counteradaptations to sexual selection and conflict. The findings also not only have implications for sexual conflict evolution, but also for conservation biology and speciation (Arnqvist et al. 2000; Holman and Kokko 2013; Gavrilets 2014). As a final note, in light of our results, we contend that applying the geographic mosaic of coevolution's perspective (Thompson 2005) to the realm of intra‐specific sexual interactions (Gosden and Svensson 2008), and in particular to the study of sexually antagonistic coevolution, would bring important insights.

## CONFLICT OF INTEREST

The authors declare no conflict of interest.

## AUTHOR CONTRIBUTIONS

E.R.E. was associated with methodology, formal analysis, investigation, review and editing of the manuscript, and visualization. F.G.G. was associated with conceptualization, methodology, formal analysis, investigation, resources, review and editing of the manuscript, visualization, supervision, project administration, and funding acquisition.

## DATA ARCHIVING

Data, files with the simulation structure and sampling protocol for the calculation of effective population sizes, and R scripts are available from the Dryad Digital Repository at https://doi.org/10.5061/dryad.r2280gbd9. The data are supplied by the authors with the request that future users of the data are aware that the experimental evolution study represents an ongoing research program.

Associate Editor: R. Snook

## Supporting information


**Table A1**. Initial and final sample sizes relative to the number of individuals assayed in the analyses of longevity and lifetime reproductive success (LRS) across experiments.
**Table A2**. Average effective population sizes and CIs (in brackets) in the different selection regimes, calculated following simulations (see text).
**Table A3**. *N*e variation in polygamous populations based on number of mating partners (2 or 3) per female, and on the correction for oviposition between matings (i.e., accounting for fecundity during mating intervals).
**Table A4**. Effect of the observed rates of infertile matings on *N*e.
**Table A5**. Means, standard deviation (S.D.) and standard error (S.E) for the response variables (longevity, in days; LRS: number of adult offspring), for the different selection regimes across experiments.
**Table A6**. Baseline Longevity.
**Figure A1**. Outline of the experimental design to measure male harm and female resistance after single mating.
**Figure A2**. Outline of the assays to measure female longevity when females were given multiple but controlled opportunities for mating/remating.
**Figure A3**. Outline of the experiment to measure male harm and female resistance after lifelong cohabitation with individuals from the other sex.
**Figure A4**. Relationship between female body size and intrinsic female longevity (measured for virgin individuals) across females from selection regimes differing in selection associated with mating system (A), or differing in selection associated with metapopulation structure (B).
**Figure A5**. Effects of mating system (monogamy vs. polygamy) and metapopulation structure (no vs. yes) in the evolution of focal females' LRS after single mating.
**Figure A6**. Cost of reproduction: negative relationship between LRS and longevity across females from selection regimes differing in selection associated with mating system (A), or differing in selection associated with metapopulation structure (B), in assays in which females mated singly.
**Figure A7**. Interaction of mating system (monogamy vs. polygamy) and metapopulation structure (no vs. yes) in the evolution of female resistance at generation 12 (left) and 30 (right) in assays with medium levels of sexual conflict (see text).
**Figure A8**. Relationship between female body size and female longevity across females from selection regimes differing in selection associated with mating system (A) or differing in selection associated with metapopulation structure (B) in assays with medium levels of sexual conflict.
**Figure A9**. Measurement of elytron length.

## References

[evl3244-bib-0001] Arbuthnott, D. , E. M. Dutton , A. F. Agrawal , and H. D. Rundle . 2014. The ecology of sexual conflict: ecologically dependent parallel evolution of male harm and female resistance in *Drosophila melanogaster* . Ecol. Lett. 17:221–228. 24215269 10.1111/ele.12222

[evl3244-bib-0002] Arnqvist, G. 2020. Mixed models offer no freedom from degrees of freedom. TREE 35:329–335. 31982147 10.1016/j.tree.2019.12.004

[evl3244-bib-0003] Arnqvist, G. , M. Edvardsson , U. Friberg , and T. Nilsson . 2000. Sexual conflict promotes speciation in insects. Proc. Natl Acad. Sci. 97:10460–10464. 10984538 10.1073/pnas.97.19.10460 PMC27046

[evl3244-bib-0004] Arnqvist, G. , and L. Rowe . 2002. Antagonistic coevolution between the sexes in a group of insects. Nature 415:787–789. 11845208 10.1038/415787a

[evl3244-bib-0005] Arnqvist, G. , and L. Rowe . 2005. Sexual conflict. Princeton University Press, Princeton.

[evl3244-bib-0006] Barr, D. J. , R. Levy , C. Scheepers , and H. J. Tily . 2013. Random effects structure for confirmatory hypothesis testing: keep it maximal. J. Mem. Lang. 68:255–278. 10.1016/j.jml.2012.11.001 PMC3881361 24403724

[evl3244-bib-0007] Bates, D. , M. Mächler , B. Bolker , and S. Walker . 2015. Fitting linear mixed‐effects models using lme4. J. Stat. Softw. 67:1–48.

[evl3244-bib-0008] Berg, E. C. , and A. A. Maklakov . 2012. Sexes suffer from suboptimal lifespan because of genetic conflict in a seed beetle. Proc. R. Soc. Lond. B 279:4296–4302. 10.1098/rspb.2012.1345 PMC3441075 22915670

[evl3244-bib-0009] Berger, D. , I. Martinossi‐Allibert , K. Grieshop , M. I. Lind , A. A. Maklakov , and G. Arnqvist . 2016. Intralocus sexual conflict and the tragedy of the commons in seed beetles. Am. Nat. 188:E98–E112. 27622882 10.1086/687963

[evl3244-bib-0010] Bilde, T. , U. Friberg , A. A. Maklakov , J. D. Fry , and G. Arnqvist . 2008. The genetic architecture of fitness in a seed beetle: assessing the potential for indirect genetic benefits of female choice. BMC Evol. Biol. 8:295. 18950531 10.1186/1471-2148-8-295 PMC2596129

[evl3244-bib-0011] Bonsall, M. B. , D. R. French , and M. P. Hassell . 2002. Metapopulation structures affect persistence of predator‐prey interactions. J. Anim. Ecol. 71:1075–1084.

[evl3244-bib-0012] Canal, D. , F. Garcia‐Gonzalez , and L. Z. Garamszegi . 2021. Experimentally constrained early reproduction shapes life history trajectories and behaviour. Sci. Rep. 11:4442. 33627681 10.1038/s41598-021-83703-1 PMC7904952

[evl3244-bib-0013] Carazo, P. , C. K. Tan , F. Allen , S. Wigby , and T. Pizzari . 2014. Within‐group male relatedness reduces harm to females in *Drosophila* . Nature 505:672–675. 24463521 10.1038/nature12949 PMC5768239

[evl3244-bib-0014] Cayetano, L. , A. A. Maklakov , R. C. Brooks , and R. Bonduriansky . 2011. Evolution of male and female genitalia following release from sexual selection. Evolution. 65:2171–2183. 21790567 10.1111/j.1558-5646.2011.01309.x

[evl3244-bib-0015] Collet, J. , D. S. Richardson , K. Worley , and T. Pizzari . 2012. Sexual selection and the differential effect of polyandry. Proc. Natl Acad. Sci. 109:8641–8645. 22592795 10.1073/pnas.1200219109 PMC3365207

[evl3244-bib-0016] De Lisle, S. P. , D. Goedert , A. M. Reedy , and E. I. Svensson . 2018. Climatic factors and species range position predict sexually antagonistic selection across taxa. Phil. Trans. R. Soc. Lond. B 373:20170415. 30150216 10.1098/rstb.2017.0415 PMC6125731

[evl3244-bib-0017] Dougherty, L. R. , E. van Lieshout , K. B. McNamara , J. A. Moschilla , G. Arnqvist , and L. W. Simmons . 2017. Sexual conflict and correlated evolution between male persistence and female resistance traits in the seed beetle *Callosobruchus maculatus* . Proc. R. Soc. Lond. B 284:20170132. 10.1098/rspb.2017.0132 PMC5454259 28539510

[evl3244-bib-0018] Eldakar, O. T. , M. J. Dlugos , J. W. Pepper , and D. S. Wilson . 2009. Population structure mediates sexual conflict in water striders. Science 326:816. 19892974 10.1126/science.1180183

[evl3244-bib-0019] Evans, J. P. , and F. Garcia‐Gonzalez . 2016. The total opportunity for sexual selection and the integration of pre‐ and post‐mating episodes of sexual selection in a complex world. J. Evol. Biol. 29:2338–2361. 27520979 10.1111/jeb.12960

[evl3244-bib-0020] Faria, G. S. , A. Gardner , and P. Carazo . 2020. Kin discrimination and demography modulate patterns of sexual conflict. Nat. Ecol. E 4:1141–1148. 10.1038/s41559-020-1214-6 PMC7610387 32451427

[evl3244-bib-0021] Faria, G. S. , S. A. M. Varela , and A. Gardner . 2015. Sex‐biased dispersal, kin selection and the evolution of sexual conflict. J. Evol. Biol. 28:1901–1910. 26190034 10.1111/jeb.12697

[evl3244-bib-0022] Fox, C. W. , M. L. Bush , D. A. Roff , and W. G. Wallin . 2004. Evolutionary genetics of lifespan and mortality rates in two populations of the seed beetle, *Callosobruchus maculatus* . Heredity 92:170–181. 14735137 10.1038/sj.hdy.6800383

[evl3244-bib-0023] Fox, C. W. , and F. J. Messina . 2018. Evolution of larval competitiveness and associated life‐history traits in response to host shifts in a seed beetle. J. Evol. Biol. 31:302–313. 29220874 10.1111/jeb.13222

[evl3244-bib-0024] Garcia‐Gonzalez, F. 2004. Infertile matings and sperm competition: the effect of “Nonsperm Representation” on intraspecific variation in sperm precedence patterns. Am. Nat. 164:457–472. 15459878 10.1086/423987

[evl3244-bib-0025] Garcia‐Gonzalez, F. , and D. Dowling . 2015. Transgenerational effects of sexual interactions and sexual conflict: non‐sires boost the fecundity of females in the following generation. Biol. Lett. 11:20150067. 25788486 10.1098/rsbl.2015.0067 PMC4387503

[evl3244-bib-0026] Garcia‐Gonzalez, F. , and J. P. Evans . 2011. Fertilization success and the estimation of genetic variance in sperm competitiveness. Evolution. 65:746–756. 20880262 10.1111/j.1558-5646.2010.01127.x

[evl3244-bib-0027] García‐Roa, R. , V. Chirinos , and P. Carazo . 2019. The ecology of sexual conflict: temperature variation in the social environment can drastically modulate male harm to females. Funct. Ecol. 33:681–692.

[evl3244-bib-0028] García‐Roa, R. , F. Garcia‐Gonzalez , D. W. A. Noble , and P. Carazo . 2020. Temperature as a modulator of sexual selection. Biol. Rev. 95:1607–1629. 32691483 10.1111/brv.12632

[evl3244-bib-0029] Garland, T. J. , and M. R. Rose , eds. 2009. Experimental Evolution: Concepts, Methods and Applications of Selection Experiments. University of California Press, Los Angeles.

[evl3244-bib-0030] Gavrilets, S. 2000. Rapid evolution of reproductive barriers driven by sexual conflict. Nature 403:886–889. 10706284 10.1038/35002564

[evl3244-bib-0031] Gavrilets, S. 2014. Is Sexual Conflict an “Engine of Speciation”? Cold Spring Harb. Perspect. Biol. 6:a017723. 25395295 10.1101/cshperspect.a017723 PMC4292158

[evl3244-bib-0032] Gay, L. , P. E. Eady , R. Vasudev , D. J. Hosken , and T. Tregenza . 2009. Does reproductive isolation evolve faster in larger populations via sexually antagonistic coevolution? Biol. Lett. 5:693–696. 19364716 10.1098/rsbl.2009.0072 PMC2781944

[evl3244-bib-0033] Gay, L. , D. J. Hosken , P. Eady , R. Vasudev , and T. Tregenza . 2010. The evolution of harm—Effect of sexual conflicts and population size. Evolution. 65:725–737. 21050188 10.1111/j.1558-5646.2010.01181.x

[evl3244-bib-0034] Gosden, T. P. , and E. I. Svensson . 2008. Spatial and temporal dynamics in a sexual selection mosaic. Evolution. 62:845–856. 18194470 10.1111/j.1558-5646.2008.00323.x

[evl3244-bib-0035] Hanski, I. 1998. Metapopulation dynamics. Nature 396:41–49.

[evl3244-bib-0036] Hanski, I. 1999. Metapopulation Ecology. Oxford University Press, Oxford.

[evl3244-bib-0037] Hanski, I. , and O. Gaggiotti . 2004. Metapopulation biology: past, present, and future. Pp. 3–22 *in* I. Hanski , and O. E. Gaggiotti , eds. Ecology, Genetics and Evolution of Metapopulations. Elsevier.

[evl3244-bib-0038] Hanski, I. , T. Mononen , and O. Ovaskainen . 2011. Eco‐evolutionary metapopulation dynamics and the spatial scale of adaptation. Am. Nat. 177:29–43. 21090992 10.1086/657625

[evl3244-bib-0039] Hassell, M. P. , R. M. May , S. W. Pacala , and P. L. Chesson . 1991. The persistence of host‐parasitoid associations in patchy environments. I. A general criterion. Am. Nat. 138:568–583. 10.1038/344150a0 2103109

[evl3244-bib-0040] Holland, B. , and W. R. Rice . 1998. Chase‐away sexual selection: antagonistic seduction versus resistance. Evolution. 52:1–7. 28568154 10.1111/j.1558-5646.1998.tb05132.x

[evl3244-bib-0041] Holland, B. , and W. R. Rice . 1999. Experimental removal of sexual selection reverses intersexual antagonistic coevolution and removes a reproductive load. Proc. Natl. Acad. Sci. U.S.A. 96:5083–5088. 10220422 10.1073/pnas.96.9.5083 PMC21820

[evl3244-bib-0042] Hollis, B. , and D. Houle . 2011. Populations with elevated mutation load do not benefit from the operation of sexual selection. J. Evol. Biol. 24:1918–1926. 21658188 10.1111/j.1420-9101.2011.02323.x PMC3156275

[evl3244-bib-0043] Hollis, B. , D. Houle , Z. Yan , T. J. Kawecki , and L. Keller . 2014. Evolution under monogamy feminizes gene expression in *Drosophila melanogaster* . Nat. Comm. 5:3482. 10.1038/ncomms4482 24637641

[evl3244-bib-0044] Holman, L. , and H. Kokko . 2013. The consequences of polyandry for population viability, extinction risk and conservation. Phil. Trans. R. Soc. Lond. B 368:20120053. 23339244 10.1098/rstb.2012.0053 PMC3576587

[evl3244-bib-0045] Holyoak, M. , and S. P. Lawler . 1996. Persistence of an extinction‐prone predator‐prey interaction through metapopulation dynamics. Ecology 77:1867–1879.

[evl3244-bib-0046] Hotzy, C. , and G. Arnqvist . 2009. Sperm competition favors harmful males in seed beetles. Curr. Biol. 19:404–407. 19230665 10.1016/j.cub.2009.01.045

[evl3244-bib-0047] Hotzy, C. , M. Polak , J. L. Rönn , and G. Arnqvist . 2012. Phenotypic engineering unveils the function of genital morphology. Curr. Biol. 22:2258–2261. 23103188 10.1016/j.cub.2012.10.009

[evl3244-bib-0048] Huffaker, C. B. , K. P. Shea , and S. G. Herman . 1963. Experimental studies on predation: complex dispersion and levels of food in an acarine predator–prey interaction. Hilgardia 34:305–329.

[evl3244-bib-0049] Kawecki, T. J. , R. E. Lenski , D. Ebert , B. Hollis , I. Olivieri , and M. C. Whitlock . 2012. Experimental evolution. TREE 27:547–560. 22819306 10.1016/j.tree.2012.06.001

[evl3244-bib-0050] Kokko, H. , and R. Brooks . 2003. Sexy to die for? Sexual selection and the risk of extinction. Ann. Zool. Fenn. 40:207–219.

[evl3244-bib-0051] Le Galliard, J. F. , P. S. Fitze , R. Ferriere , and J. Clobert . 2005. Sex ratio bias, male aggression, and population collapse in lizards. Proc. Natl Acad. Sci. 102:18231–18236. 16322105 10.1073/pnas.0505172102 PMC1312374

[evl3244-bib-0052] Levin, S. A. 1974. Dispersion and population interactions. Am. Nat. 108:207–228.

[evl3244-bib-0053] Long, T. A. F. , A. F. Agrawal , and L. Rowe . 2012. The effect of sexual selection on offspring fitness depends on the nature of genetic variation. Curr. Biol. 22:204–208. 22226747 10.1016/j.cub.2011.12.020

[evl3244-bib-0054] Łukasiewicz, A. , A. Szubert‐Kruszynska , and J. Radwan . 2017. Kin selection promotes female productivity and cooperation between the sexes. Sci. Adv. 3:e1602262. 28345048 10.1126/sciadv.1602262 PMC5351977

[evl3244-bib-0055] Lymbery, S. J. , and L. W. Simmons . 2017. Males harm females less when competing with familiar relatives. Proc. R. Soc. Lond. B 284:20171984. 10.1098/rspb.2017.1984 PMC5719177 29142115

[evl3244-bib-0056] Lymbery, S. J. , B. Wyber , J. L. Tomkins , and L. W. Simmons . 2020. No evidence for divergence in male harmfulness or female resistance in response to changes in the opportunity for dispersal. J. Evol. Biol. 33:966–978. 32279381 10.1111/jeb.13628

[evl3244-bib-0057] Maklakov, A. A. , L. Cayetano , R. C. Brooks , and R. Bonduriansky . 2010. The roles of life‐history selection and sexual selection in the adaptive evolution of mating behavior in a beetle. Evolution. 64:1273–1282. 19930453 10.1111/j.1558-5646.2009.00904.x

[evl3244-bib-0058] Martin, O. Y. , and D. J. Hosken . 2003. The evolution of reproductive isolation through sexual conflict. Nature 423:979–982. 12827200 10.1038/nature01752

[evl3244-bib-0059] Martin, O. Y. , and D. J. Hosken . 2004. Reproductive consequences of population divergence through sexual conflict. Curr. Biol. 14:906–910. 15186748 10.1016/j.cub.2004.04.043

[evl3244-bib-0060] Martin, O. Y. , D. J. Hosken , and P. I. Ward . 2004. Post‐copulatory sexual selection and female fitness in *Scathophaga stercoraria* . Proc. R. Soc. Lond. B 271:353–359. 10.1098/rspb.2003.2588 PMC1691601 15101693

[evl3244-bib-0061] McDonald, G. C. , R. James , J. Krause , and T. Pizzari . 2013. Sexual networks: measuring sexual selection in structured, polyandrous populations. Phil. Trans. R. Soc. Lond. B 368:20120356. 23339246 10.1098/rstb.2012.0356 PMC3576589

[evl3244-bib-0062] Messina, F. J. , and J. D. Fry . 2003. Environment‐dependent reversal of a life history trade‐off in the seed beetle *Callosobruchus maculatus* . J. Evol. Biol. 16:501–509. 14635850 10.1046/j.1420-9101.2003.00535.x

[evl3244-bib-0063] Messina, F. J. , and A. F. Slade . 1999. Expression of a life‐history trade‐off in a seed beetle depends on environmental context. Physiol. Entomol. 24:358–363.

[evl3244-bib-0064] Morimoto, J. , G. C. McDonald , E. Smith , D. T. Smith , J. C. Perry , T. Chapman , et al. 2019. Sex peptide receptor‐regulated polyandry modulates the balance of pre‐ and post‐copulatory sexual selection in Drosophila. Nat. Comm. 10:283. 10.1038/s41467-018-08113-w PMC6336784 30655522

[evl3244-bib-0065] Parker, G. A. 1979. Sexual selection and sexual conflict. Pp. 123–166 *in* M. S. Blum , and N. A. Blum , eds. Sexual Selection and Reproductive Competition in Insects. Academic Press, New York.

[evl3244-bib-0066] Perry, J. C. , C. J. Garroway , and L. Rowe . 2017. The role of ecology, neutral processes and antagonistic coevolution in an apparent sexual arms race. Ecol. Lett. 20:1107–1117. 28683517 10.1111/ele.12806

[evl3244-bib-0067] Perry, J. C. , and L. Rowe . 2018. Sexual conflict in its ecological setting. Phil. Trans. R. Soc. Lond. B 373:20170418. 30150217 10.1098/rstb.2017.0418 PMC6125725

[evl3244-bib-0068] Pitnick, S. , and F. Garcia‐Gonzalez . 2002. Harm to females increases with male body size in *Drosophila melanogaster* . Proc. R. Soc. Lond. B 269:1821–1828. 10.1098/rspb.2002.2090 PMC1691094 12350270

[evl3244-bib-0069] Pizzari, T. , J. M. Biernaskie , and P. Carazo . 2015. Inclusive fitness and sexual conflict: how population structure can modulate the battle of the sexes. Bioessays 37:155–166. 25389109 10.1002/bies.201400130

[evl3244-bib-0070] Rankin, D. J. , K. Bargum , and H. Kokko . 2007. The tragedy of the commons in evolutionary biology. TREE 22:643–651. 17981363 10.1016/j.tree.2007.07.009

[evl3244-bib-0071] Rankin, D. J. , U. Dieckmann , and H. Kokko . 2011. Sexual conflict and the tragedy of the commons. Am. Nat. 177:780–791. 21597254 10.1086/659947

[evl3244-bib-0072] Rodrigues, L. R. , M. Torralba Sáez , J. Alpedrinha , S. Lefèvre , M. Brengues , S. Magalhães , et al. 2021. Consequences of population structure for sex allocation and sexual conflict. J. Evol. Biol. 34:525–536. 33314358 10.1111/jeb.13755

[evl3244-bib-0073] Rodriguez‐Exposito, E. 2018. Evolutionary responses to the independent and interacting action of sexual selection and population spatial structure: Insights from experimental evolution in a species with sexual conflict. PhD Thesis, Universidad de La Laguna.

[evl3244-bib-0074] Roff, D. A. 2002. Life‐history evolution. Sinauer Associates, Sunderland, MA.

[evl3244-bib-0075] Ronn, J. , M. Katvala , and G. Arnqvist . 2006. The costs of mating and egg production in *Callosobruchus* seed beetles. Anim. Behav. 72:335–342.

[evl3244-bib-0076] Rowe, L. , and T. Day . 2006. Detecting sexual conflict and sexually antagonistic coevolution. Phil. Trans. R. Soc. Lond. B 361:277–285. 16612887 10.1098/rstb.2005.1788 PMC1569602

[evl3244-bib-0077] Saccheri, I. , and I. Hanski . 2006. Natural selection and population dynamics. TREE 21:341–347. 16769435 10.1016/j.tree.2006.03.018

[evl3244-bib-0078] Savalli, U. M. , and C. W. Fox . 1999. The effect of male mating history on paternal investment, fecundity and female remating in the seed beetle *Callosobruchus maculatus* . Funct. Ecol. 13:169–177.

[evl3244-bib-0079] Sayadi, A. , A. Martinez Barrio , E. Immonen , J. Dainat , D. Berger , C. Tellgren‐Roth , et al. 2019. The genomic footprint of sexual conflict. Nat. Ecol. E 3:1725–1730. 10.1038/s41559-019-1041-9 31740847

[evl3244-bib-0080] Schielzeth, H. 2010. Simple means to improve the interpretability of regression coefficients. Methods Ecol. E 1:103–113.

[evl3244-bib-0081] Schielzeth, H. , and W. Forstmeier . 2009. Conclusions beyond support: overconfident estimates in mixed models. Behav. Ecol. 20:416–420. 19461866 10.1093/beheco/arn145 PMC2657178

[evl3244-bib-0082] Simmons, L. W. , and F. Garcia‐Gonzalez . 2008. Evolutionary reduction in testes size and competitive fertilization success in response to the experimental removal of sexual selection in dung beetles. Evolution. 62:2580–2591. 18691259 10.1111/j.1558-5646.2008.00479.x

[evl3244-bib-0083] Smith, D. T. , N. V. Clarke , J. M. Boone , C. Fricke , and T. Chapman . 2017. Sexual conflict over remating interval is modulated by the sex peptide pathway. Proc. R. Soc. B‐Biol. Sci. 284:20162394. 10.1098/rspb.2016.2394 PMC5360916 28250180

[evl3244-bib-0084] Thompson, J. N. 2005. The Geographic Mosaic of Coevolution. University of Chicago Press, Chicago, IL.

[evl3244-bib-0085] Tilman, D. 1994. Competition and biodiversity in spatially structured habitats. Ecology 75:2–16.

[evl3244-bib-0086] Travers, L. M. , F. Garcia‐Gonzalez , and L. W. Simmons . 2015. Live fast die young life history in females: evolutionary trade‐off between early life mating and lifespan in female *Drosophila melanogaster* . Sci. Rep. 5:15469. 26482533 10.1038/srep15469 PMC4612512

[evl3244-bib-0087] Walsh, J. B. , and M. Lynch . 2018. Evolution and Selection of Quantitative Traits. Oxford University Press, Oxford, UK.

[evl3244-bib-0088] Wigby, S. , and T. Chapman . 2005. Sex peptide causes mating costs in female *Drosophila melanogaster* . Curr. Biol. 15:316–321. 15723791 10.1016/j.cub.2005.01.051

[evl3244-bib-0089] Wild, G. , T. Pizzari , and S. A. West . 2011. Sexual conflict in viscous populations: the effect of the timing of dispersal. Theor. Popul. Biol. 80:298–316. 10.1016/j.tpb.2011.09.002 21982746

[evl3244-bib-0090] Williams, G. C. 1966. Natural selection, the cost of reproduction, and a refinement of lack's principle. Am. Nat. 100:687–690.

[evl3244-bib-0091] Yasui, Y. , and F. Garcia‐Gonzalez . 2016. Bet‐hedging as a mechanism for the evolution of polyandry, revisited. Evolution. 70:385–397. 26748458 10.1111/evo.12847

[evl3244-bib-0092] Zajitschek, S. R. K. , D. K. Dowling , M. L. Head , E. Rodriguez‐Exposito , and F. Garcia‐Gonzalez . 2018. Transgenerational effects of maternal sexual interactions in seed beetles. Heredity 121:282–291. 29802349 10.1038/s41437-018-0093-y PMC6082829

[evl3244-bib-0093] Zuur, A. , E. N. Ieno , N. Walker , A. A. Saveliev , and G. M. Smith . 2009. Mixed Effects Models and Extensions in Ecology with R. Springer, New York.

