# Supplementary material for: Metapopulation structure modulates sexual antagonism
Source: Evol Lett. 2021 Jun 30;5(4):344–58. doi: 10.1002/evl3.244 (PMC8327942; doi:10.1002/evl3.244)
Supplement: Supplementary file 1 — Table A1. Initial and final sample sizes relative to the number of individuals assayed in the analyses of longevity and lifetime reproductive success (LRS) across experiments. Table A2. Average effective population sizes and CIs (in brackets) in the different selection regimes, calculated following simulations (see text). Table A3. Ne variation in polygamous populations based on number of mating partners (2 or 3) per female, and on the correction for oviposition between matings (i.e., accounting for fecundity during mating intervals). Table A4. Effect of the observed rates of infertile matings on Ne. Table A5. Means, standard deviation (S.D.) and standard error (S.E) for the response variables (longevity, in days; LRS: number of adult offspring), for the different selection regimes across experiments. Table A6. Baseline Longevity. Figure A1. Outline of the experimental design to measure male harm and female resistance after single mating. Figure A2. Outline of the assays to measure female longevity when females were given multiple but controlled opportunities for mating/remating. Figure A3. Outline of the experiment to measure male harm and female resistance after lifelong cohabitation with individuals from the other sex. Figure A4. Relationship between female body size and intrinsic female longevity (measured for virgin individuals) across females from selection regimes differing in selection associated with mating system (A), or differing in selection associated with metapopulation structure (B). Figure A5. Effects of mating system (monogamy vs. polygamy) and metapopulation structure (no vs. yes) in the evolution of focal females' LRS after single mating. Figure A6. Cost of reproduction: negative relationship between LRS and longevity across females from selection regimes differing in selection associated with mating system (A), or differing in selection associated with metapopulation structure (B), in assays in which females mated singly. Figure A7. Interaction o [file EVL3-5-344-s001.pdf]

Appendix

Metapopulation structure modulates sexual antagonism

E. Rodriguez-Exposito[1,†], F. Garcia-Gonzalez[1,2*]

[1]Doñana Biological Station (EBD-CSIC), Calle Americo Vespucio 26, Isla de la Cartuja 41092, Sevilla, Spain.
[2]Centre for Evolutionary Biology, School of Biological Sciences, University of Western Australia, 35 Stirling Highway, Crawley 6009, Western Australia, Australia.
†Currently at Institute of Natural Products and Agrobiology (IPNA-CSIC). Avenida Astrofisico Francisco Sánchez, 3, San Cristóbal de La Laguna 38206, Santa Cruz de Tenerife, Spain. Email address: edroex@gmail.com

*Correspondence to: paco.garcia@ebd.csic.es

Table of Contents

Additional details on methods **2**
Study system 2
Additional details on the propagation of selection lines 2
Generation and use of tester individuals 3
Body size measurements 4
Effective population sizes (N_e) 4
Full details for statistical analyses 7

Additional results **9**
Extended results on effective population sizes (N_e) 9
Baseline longevity 9
Extended results from the extreme levels of sexual conflict assay 9

Appendix Tables **11**
Table A1. 11
Table A2. 12
Table A3. 13
Table A4. 14
Table A5. 15
Table A6. 17

Appendix Figures **18**
Figure A1. 18
Figure A2. 19
Figure A3. 20
Figure A4. 21
Figure A5. 22
Figure A6. 23
Figure A7. 24
Figure A8. 25
Figure A9. 26

References **27**

Additional details on methods

Study system

The seed beetle *Callosobruchus maculatus* is a widely distributed pest of stored legumes. Adults are facultatively aphagous (they do not need to feed or drink) and the species is a capital breeder since the beetles obtain all of the resources needed for survival and reproduction during the larval stage (see below and Messina and Fry 2003). Mated females cement their eggs on the surface of the host beans. Upon hatching the larvae enter the seed, where they complete their development and pupate, before emerging as adults. The species has a short life cycle (around 22 days at 30◦C) and high fecundity (a female typically produces several scores of eggs over the course of a few days). The species is a model system in the study of sexual conflict (Crudgington and Siva-Jothy 2000; Arnqvist et al. 2005; Gay et al. 2010; Cayetano et al. 2011; Zuk et al. 2014; Berger et al. 2016; Dougherty and Simmons 2017; Iglesias-Carrasco et al. 2018a; Sayadi et al. 2019; McNamara et al. 2020). The species is highly polygamous and the male intromittent organ is densely coated with thick spines. These spines determine to a large extent male reproductive success and reduce female fitness (Crudgington and Siva-Jothy 2000; Hotzy and Arnqvist 2009; Gay et al. 2010; Hotzy et al. 2012). We sourced the beetles from an outbred population (South Indian stock population) that presents substantial phenotypic and genetic variance (Fox et al. 2004; Bilde et al. 2008; Berg and Maklakov 2012; Rodriguez-Exposito 2018; Zajitschek et al. 2018; Canal et al. 2021). Beetles were kept in walk-in climate chambers (Fitoclima 10000 EHF, Aralab) at a constant 29 °C temperature with 40% humidity and a 12 h/12 h light/dark cycle, and were cultured in organic mung beans (*Vigna radiata*). Further details about the culturing of our stock population can be found elsewhere (Zajitschek et al. 2018; Canal et al. 2021). Importantly, the standard culturing conditions of *C. maculatus* resemble the conditions to which these animals have adapted for thousands of years (infestation of dry legume seed storages), and the conditions that natural populations experience nowadays, which makes this species a suitable laboratory model system (Berger et al. 2014).

Additional details on the propagation of selection lines

The selection of inoculated beans from each line to generate the next generation of animals was carried out randomly but to avoid unintended selection we followed a systematized plan for the propagation of the lines. This plan also ensured that we imposed the appropriate levels of sexual interactions envisaged by the experimental design:

a) Non-structure and polygamy (*NSPoly*) lines: The 25 breeding couples per line were placed in a 750 ml plastic container with approx. 1600 beans (64 seeds per female). As for all selection lines, sexual interactions and egg laying took place during two days (see main text). For each line, the 50 breeders for the next generation were randomly selected from the virgin adults emerging from the 150 inoculated beans randomly collected from the population container.

b) Structure and polygamy (*SPoly*) lines: For each of the 5 subpopulations within each line we placed five breeding couples in a 140 ml plastic container with approx. 320 beans (64 seeds per female). Thirty inoculated beans per subpopulation (i.e., 150 per line) were randomly selected and individually kept. A random selection of 4 virgin males and 4 virgin females emerged from these beans in each subpopulation plus one virgin adult of each sex emerging from the beans in a different subpopulation (imposed migration, see above and Fig. 1) were designated the subpopulation breeders for the next generation. Thus, within each line metapopulation structure was mimicked (with gene flow among subpopulations) and individuals could engage in polygamous sexual interactions.

c) Non-structure and monogamy (*NSMono*) lines: The 25 breeding couples per line

were each placed in a 30 ml plastic container with 64 beans (i.e., 64 beans per female). Six inoculated beans per container (i.e., 150 beans per line, each bean containing a single egg) were randomly selected. The breeders for the next generation were then randomly sourced from the virgin adults emerging from the pool of 150 beans collected in each line. Thus, within each of these lines, monogamous matings were enforced and there was no population subdivision whatsoever (breeders originated from the pool of 150 beans).

d) Structure and monogamy (*SMono* lines). In this treatment, as in *SPoly* lines, there were 5 breeding pairs for each of the 5 subpopulations in each line. However, monogamy was enforced because each male-female pair was housed in a 30 ml plastic container (with 64 beans each). Six inoculated beans per container were randomly collected, as in the *NSMono* lines; however, to impose the effects of spatial population structure, inoculated beans were not pooled at the population level (as in *NSMono* lines) but at the subpopulation level (n = 30 inoculated beans per subpopulation, n beans per line = 150). Like *SPoly* lines, a random selection of 4 virgin males and 4 virgin females emerged from the beans in each subpopulation plus one virgin adult of each sex emerging from the beans in a different subpopulation (imposed migration, see above and Fig. 1) were designated the subpopulation breeders for the next generation. Therefore, within each of these lines, metapopulation structure was mimicked (with gene flow among subpopulations) and the scope for sexual selection and sexual conflict was greatly reduced (individuals could only engage in enforced monogamous sexual interactions).

Generation and use of tester individuals

Each of the near-isogenic lines was started with one founder male-female pair from the stock population and the line was propagated through a full brother-full sister mating protocol that spanned many generations (>15, see specific details about the number of generations in each case below). In some cases the assays were carried out using tester individuals from such pure near-isogenic lines. In other cases, tester individuals were obtained after crossing two near-isogenic lines. These crosses were carried out to generate a standardized but heterozygous genetic background (see for instance Garcia-Gonzalez and Dowling 2015). We refer to the lines from these crosses as Standardized Heterozygous Lines (SHL). Crosses leading to SHL lines were established by housing together in a 750 ml plastic container 25 males and 25 females collected from two randomly chosen near-isogenic lines (12 beetles from each sex from one of the lines, and 13 beetles from each sex from the other line) two generations before carrying out the assays. These lines were kept with a non-overlapping generations protocol in which within-line matings (25 males and 25 females) were allowed. Likewise, when pure near-isogenic lines were used, two generations before the assays 25 pairs from each line were transferred to 750 ml containers and within-line matings allowed under a non-overlapping generations protocol. This punctual "mass-breeding" within-line protocol ensured the production of enough tester animals for the assays. Near-isogenic lines or SHLs were used depending on logistics, to obtain enough tester animals for the assays, but pure near isogenic-lines and SHLs were never mixed. We thus standardized the interacting genetic background with which experimentally evolved individuals were measured. Since the relevant comparisons in our tests are those carried out across selection regimes within each specific assay, and for any given assay the tester strain used was identical across selection regimes, the type of sourcing for tester animals does not affect the conclusions in the study. Details of the tester individuals used in the different experiments are as follows:

- Medium levels of sexual conflict assay (female longevity under variable female mating rates): Variation attributable to mate identity in female mating rates was minimized by using tester males that belonged to a same near-isogenic line or a SHL in each mating opportunity across all females. All females across all selection lines were exposed to the same line ID of tester males each mating opportunity, and the ID of the near-isogenic line or SHL used to generate the tester males were rotated across mating opportunities. Near-isogenic

lines used had been cultured on a full sib mating protocol for 15 and 33 generations, for the assays carried out at generations 12 and 30, respectively.

- Low levels of sexual conflict assay (female lifetime reproductive success -LRS- and longevity after single mating): Tester individuals were drawn from a near-isogenic line generated after 35 generations of full-sib matings.
- Extreme levels of sexual conflict assay (female LRS and longevity under continuous exposure to intense male harassment): Tester individuals used in this experiment were collected from a near-isogenic line generated after 50 generations of full-sib matings.

Body size measurements

Elytron length was used as a proxy of body size (Eady et al. 2004; Rodriguez-Exposito 2018). Elytron length was measured from photographs of frozen beetles. Specimens were placed on top of a pedestal made of Blu Tack (Bostik, US)(Fig. A9) and images were taken using a stereomicroscope SteREO Discovery V8 (Carl Zeiss Microscopy GmbH, Germany) connected to an AxioCam Icc 1 camera (Carl Zeiss, Germany). The maximum length of the right elytron was measured on the images (Fig. A9) using the software ZEN 2 (blue edition, Carl Zeiss, Germany, 2011). Body size was measured on all male or female focal individuals. The size of the tester individuals was also measured in the single mating assays. The repeatability of body size measures, as calculated from subsets of individuals that were measured twice, is very high. All the experimental females in the female mating frequency assays were measured twice, and the repeatability of elytron length, calculated following Becker (1984), was 0.99 and 0.98 for generations 12 ($n = 112$ females) and 30 ($n = 160$ females) (all $p < 0.0001$). In the assays of sexual conflict following single mating the elytron length of 319 males and 317 females was measured twice and repeatabilities were again high (males: 0.99, females: 0.93; all p-values <0.0001). The repeatability of elytron length was additionally measured in a set of 320 males and 320 females at generation 34; repeatabilities were very high regardless of sex (all $R> 0.99$; all $p < 0.0001$). In light of these results, the rest of individuals were only measured once, and for those individuals with two measures of body size we used the mean value of these two measurements in the analyses.

Effective population sizes (N_e)

Under our experimental protocol, slight variation in effective population sizes due to spatial structuring may arise due to sampling. Larger variation in N_e is, however, expected between polygamous and monogamous populations due to a series of reasons including sexual selection. Apart from the fact that in polygamous populations genetic hitchhiking of genetic polymorphisms in linkage disequilibrium with sexually selected alleles can lead indirectly to reduced N_e and genetic diversity (Snook et al. 2009), variance in male reproductive success due to variation in mating and fertilization success under sexual selection may reduce N_e in these populations compared to monogamous lines. In contrast, a higher reduction in N_e would be expected to arise in monogamous populations if infertile mating rates, due to either male or female infertility, were common. Infertile matings would reduce N_e in monogamous populations to a larger extent than in polygamous populations because polyandry would buffer the outcome of male infertility on female productivity (Garcia-Gonzalez 2004; Yasui and Garcia-Gonzalez 2016). For instance, a monogamous pair in which the male is infertile results in that neither the male nor the female leaves offspring, while under polyandry only high rates of infertility among males would result in the females having null reproductive success because the chances that all mates of a polyandrous female are all infertile would be low (Garcia-Gonzalez 2004). In other words, the more mates a female mates with, the less likely it is for the female to have complete reproductive failure (Yasui and Garcia-Gonzalez 2016). Our lines do not differ in the extent of infertility and the average infertile matings rate is 2.54% (Rodriguez-Exposito and Garcia-Gonzalez, unpublished; Rodriguez-Exposito 2018). This means that, on average, for monogamous lines, less than one pair of beetles out of the 25 pairs in each population would produce no

offspring (probability that a monogamous female produces no offspring = 0.025; 0.63 females out of the 25 in each line). Assuming that all infertile matings are the result of male infertility, the probability that any polyandrous female produces no offspring would be, however, 0.00064 (i.e., only 0.16 females out of the 25 in each line), if she mates with two males, or 0.000016 (0.0004 females out of the 25 in each line), if she mates with three males.

Assuming that male reproductive success is random the effective population size can be calculated as:

$$Ne = \frac{4\,Nf\;Nm}{(Nf+Nm)} \qquad (1)$$

where *Nf* is the number of breeding females and *Nm* is the number of breeding males (Crow and Kimura 1970; Falconer and Mackay 1996; Snook et al. 2009; Walsh and Lynch 2018). Incorporating female multiple mating and variance in male reproductive success, the effective population size can be calculated as (Rice and Holland 2005; Snook et al. 2009):

$$Ne = \frac{4\,NfNf}{Nf\sum_{1}^{i} P_i{}^2+Nf} \qquad (2)$$

where, P_i is the proportion of offspring sired by male *P* (Rice and Holland 2005). However, this formula, which has been widely used across studies, applies to situations in which each female is mated to a different set of males. Our case is more complex because in the polygamous populations each female can mate with different males but each male can also mate with different females. Furthermore, in some populations we have structure in regards to mating interactions. In our case, therefore, we recurred to Monte Carlo simulations to approximate effective population sizes. We used Poptools 3.06 (Hood 2008) to mimic the conditions in each selection regime, and to draw estimates of N_e for each selection regime. For each selection regime and each combination of conditions (scenarios) simulated, we calculated the number of sires and dams represented in a given generation. We then calculated N_e summing up both the number of sires and dams. The number of iterations within each scenario was set to 10000. The average and 95% CIs for N_e estimations for all the scenarios within each selection regime are shown below. The files with the simulation structure and sampling protocol from Poptools can be found on Dryad at https://doi.org/10.5061/dryad.r2280gbd9.

Simulations needed to be adjusted for each selection regime to mimic the precise extraction protocol to obtain the breeding individuals followed in our selection experiment:

a) *NSPoly* lines:

Virgin *C. maculatus* females typically mate within seconds or minutes after encountering a male, and many singly-mated females that are given the opportunity to remate with a different male 24h after their first mating do so (Rodriguez-Exposito 2018). Accordingly, and given that females in *NSPoly* lines are continuously housed with plenty of males, in the simulations we conservatively assumed that over the course of 48 hours females would mate with at least two males. We simulated two scenarios here: double and triple matings. Our estimate of N_e is probably conservative as it is likely that mating rates are higher in the polygamous populations. The distribution of paternity for each male was based on the empirical estimate that last male sperm precedence when females mate to two or three males averages 0.80 in the species (Eady 1991; Eady 1994; Eady and Tubman 1996; Simmons 2001; Vasudeva et al. 2014; McNamara et al. 2016). Fecundity is kept constant at 40 eggs in these lines and all lines, based on previous findings regarding number of eggs produced the first days after mating (Zajitschek et al. 2018). In any case, we checked that reasonable variation around fecundity did not introduce substantial variation in the results (see below). A key factor that has been overlooked in previous studies but that we implement in our calculations is that mating

interval matters for the calculation of N_e. This is so because even in a system with strong last male sperm precedence females would produce offspring of first male(s) in between matings. The production of offspring before all the known (or estimated) matings have been completed would affect N_e calculations to a great extent. We took into account this factor, and obtained estimates with or without correction for fecundity between matings. In absence of the correction the (unrealistic) assumption is that all matings take place at the same time. When the correction was applied we assumed that the first mating of females occurred at time 0 (virgins females typically mate within a few seconds or minutes after sexual encounter, as noted), and that each subsequent mating occurred 16 hours after the previous mating (i.e., for a double mating scenario the two matings would take place at time 0 and 16 h, and for a triple mating scenario the matings would take place at times 0, 16 and 32 h). Variation in mating interval does not introduce large variation in N_e (results not shown). For each case we calculated paternity shares according to the sperm precedence patterns in the system (see files with the simulation structure and sampling protocol at https://doi.org/10.5061/dryad.r2280gbd9). For instance, if a female mates with male A at time 0 h, male B at time 16 h, and male C at time 32 h, and assuming, for simplicity, constant oviposition rate over the first 48 hours (i.e, 0.83 eggs/hour for a total fecundity of 40 eggs), the distribution of paternity at the end of the 48 hours is calculated as follows: during the first 16 h (i.e., before the female's second mating) the female will lay 13 eggs (0.83 eggs per hour during 16 hours) from 1st male. For the remaining 27 eggs, 13 eggs will be laid during the next 16 hours (after the second mating and before the third mating). These 13 eggs would be sired according to P_n (paternity of the last male to mate) = 0.8; that is, 10 eggs would be sired by the second male and 3 eggs would be sired by the 1st male. The remaining eggs (14) would be laid after the female has mated with the three males, and again, they would be sired according to the sperm precedence pattern in the system. So, 11 eggs would be sired by the third male, 2 eggs from the second male, and 1 egg would be sired by the first male. In sum, the distribution of paternity for the first, second and third males, would be 17, 12, and 11 offspring, respectively. This illustrates that considering mating interval and fecundity in between matings is important. Failure to do so yields a quite different distribution of paternity. The distribution of paternity under the assumption that the eggs of a female are sired by three males according to P_n = 0.8 without considering that mating interval affects fecundity is 2, 6, and 32 offspring for the first, second and third males to mate with the female, respectively. The correction, however, does not imply a substantial change in N_e in our case (see Appendix Results). We also implemented in the simulations scenarios were infertile matings were considered and confirmed that their effects were trivial upon N_e variation (see the section: Extended results on effective population sizes).

b) *SPoly* lines:

Simulations here proceed as described above with the important exception that individuals are structured into 5 populations and that there is a 20% migration rate among subpopulations. We implemented in the simulations these features, and mimicked the empirical breeding protocol: thirty offspring are randomly extracted from each subpopulation, from which a random selection of 4 males and 4 females, plus another couple of adults randomly selected from a different subpopulation (migration) are the breeders at the subpopulation level for the next generation. This protocol is set for each of the 5 subpopulations within each line and the number of effective breeders at the line level is calculated. With these numbers, as above, we estimated N_e summing up the number of effective breeders, or using equation 1.

N_e in spatially structured populations can be also calculated following Wright's (1951) island model, where a metapopulation consists of *d* demes, each containing *N* individuals, and each deme contributes a fraction of its genes *m* (equal across demes) to the migrant pool, which is then distributed among the remaining demes (Walsh and Lynch 2018: Pp. 72-73):

$$N_e = Nd + \frac{(d-1)^2}{4dm} \qquad (3)$$

where *Nd* is the sum of the demic effective sizes.

In our case, the structured populations consist of 5 demes, each containing a fixed number of individuals (10), and each deme contributes an equal fraction of its genes (0.2%, as 2 out of the 10 individuals in each deme migrate to a different deme) to the migrant pool. We can therefore calculate the effective population size in each deme following equation 3 as if there was no variance in male reproductive success: d = 5, N_e of each deme = 10, Nd = 50, m = 0.2, Metapopulation N_e = 54. This estimate is, however, unrealistic as it does not take into account variance in male reproductive success. We applied simulations in which we implemented variance in male reproductive success (as above, with P_n = 0.8) and calculated the sum of the demic effective sizes (*Nd*) in absence of migration to then apply equation 3. Using this method we estimated N_e with and without considering mating intervals and subsequent distributions of paternity (see above), and for the case of two or three mating partners per female, as above.

c) *NSMono* lines:
In these lines each monogamous female was mated to a unique monogamous male and each pair equally contributed (with the exception of occasional infertility) with 6 offspring to the pool of offspring (n = 150) that was randomly sampled to begin the next generation. Such random extraction from the pool of 150 offspring to seed the next generation inevitably means a slight reduction in N_e values compared to a situation where exactly two individuals are taken from each couple. Thus we also applied to these lines simulations to estimate N_e following the precise protocol we followed in our selection experiment. A scenario considering the effects of infertile matings (1 infertile male out of the 25 in each population; see above) was also inspected.

d) *SMono* lines:
In the simulations to ascertain N_e in *SMono* lines we introduced, as for the *SPoly* lines, spatial structure according to the empirical protocol. Here each monogamous male-female pair contributed with 6 offspring to a pool of 30 offspring per subpopulation, from which the breeders (n = 5 pairs of breeders per subpopulation) for the next generation were randomly sampled (taking into account that 1 pair of breeders migrates from one population to another). This is simulated for each of the 5 subpopulations within each line and the number of effective breeders at the level of the line is calculated. As for *SPoly* lines, we also calculated N_e using the island model formula (equation 3), and for the different combination of conditions (variation in mating frequency, infertility and oviposition in between matings).

Full details for statistical analyses

Modelling approach:

In the analysis of fitness traits after single mating or continuous male-female cohabitation we had data on the evolution of male harm (fitness of tester females mated to focal males), and data on the evolution of female resistance (fitness of focal females mated to tester males). The response variables in this group of assays were female longevity and LRS. We also measured baseline longevity data in virgin individuals from the different selection lines. We run a series of complementary analytical approaches on the response variables. First, since the variables are count variables (days alive, number of offspring) we run analyses using Generalized Linear Mixed Models (GLMMs) with a Poisson error distribution. These models were fitted using the function *glmer* in the package *lme4* (Bates et al. 2015). To account for issues of data dispersion in our GLMM we applied quasi-likelihood by correcting the standard errors of the estimates by the dispersion factor and then recomputing Z- and p-values accordingly. Second, complementary and confirmatory Linear Mixed Models (LMMs) were fitted on the dependent

variables using the function *lmer* in the package *lme4* (Bates et al. 2015). To improve the validation of the LMM models we squared LRS. Both LMMs and GLMMs yielded similar results in general, and we show here the results pertaining to LMMs because the validation of these models was superior.

Fixed effects structure of models:

In general, as predictors, the models included the mating system experimental evolution treatment (monogamy/polygamy), metapopulation experimental evolution treatment (yes/no), and the interaction of these two factors, as fixed effects. Body size data pertaining to the individuals in the analyses were included as covariates in all the analyses, and in the analyses of responses in male harm and female resistance we also included the second order interactions between male or female body size and treatments because male body size might be associated with male harm and female body size with female resistance (see results and Pitnick and Garcia-Gonzalez 2002), and the influence of body size on harm/resistance could be expected to differ according to treatment. For instance, larger male body size should be associated with increased harm to females but only for males from polyandrous lines; larger male body size in monandrous males could, however, expected to increase female longevity if larger males transfer larger ejaculates (Czesak and Fox 2003; Iglesias-Carrasco et al. 2018b). To account for reproduction-survival relationships (see text), female longevity was included as a covariate in the analyses of LRS, while LRS was included in the models where female longevity was the response variable. In the data from continuous male-female cohabitation there was slight variation in the age that tester females entered the male harm assays (mean ± SE age of females: 1.75 ± 0.07, range 1-5 days old), and thus female age was also entered, as a control covariate, in these models. Female age was less variable in the assays where females from the selection lines were cohabiting with tester males (female resistance assays: mean ± SE age of females: 1.05 ± 0.02, range 1-2 days old), but age was also included in the models. In all models all the covariates were mean-centered (Schielzeth 2010).

In the assays in which experimental females were given controlled opportunities for remating only female longevity (number of days alive after entering the assay) was measured as in these assays females were not allowed to lay eggs. All females mated at least once and no female died before completing the mating opportunities period. Tough mating frequency does not differ among selection regimes at the time of the test (Rodriguez-Exposito and Garcia-Gonzalez, unpublished; Rodriguez-Exposito 2018), female mating frequency was included as a control covariate in the model (mean mating frequency = 1.94, SE = 0.04, range 1-4, n = 262, 73% of females mated more than once). There was very little variation in female age at the start of the assay (mean ± SE = 1.48 ± 0.04, range 1-3, n = 262), but age was nonetheless entered as another control covariate in the models, as well as generation. Predictors in these models included the two experimental evolution treatments, female body size, and the second order interactions involving these three predictors.

Random effects structure of models:

Line ID (unique code for each of the 16 selection lines) was included as a random effect in all models. Models included covariates for which interactions with the experimental evolution treatments were deemed of interest (e.g., see discussion on body size, or on the relationship between LRS and longevity above) and so we fitted by-line ID random intercept and random slopes models to avoid inflation of type I error (Schielzeth and Forstmeier 2009; Barr et al. 2013; Bates et al. 2015; Arnqvist 2020). The correlations between intercept and slopes were also included in the models (Barr et al. 2013). Nevertheless, in complex models (e.g., including 2 fixed factors plus three covariates) there were occasional issues of convergence. In such cases, the models were run without including the correlation between random intercept and random slopes, to simplify the random effects structure of the model. This decision was based

on the results of Barr et al. (2013) informing that when maximal linear mixed effects models fail to convergence, removing random correlations does not affect the anti-conservativity of the model. Models excluding random correlations are indicated in the table legends when applicable. For covariates that were included in the model as control predictors (e.g., age of female when entering the assay) random slopes were not modelled, as interactions between these control predictors and the treatments were not present in the model (they were not the focus of the analyses or justified by the data), and this procedure is expected not to inflate type I errors (Barr et al. 2013).

Significance of effects and additional considerations:

The function *Anova* (*car* package) was employed to evaluate the statistical significance of fixed effects, using Type II (Type III in the presence of significant interactions) Wald Chi-square tests. Significance of effects was carried out on maximum likelihood models, while parameter estimates were calculated using Restricted Maximum Likelihood, as it has been recommended (Zuur et al. 2009). Significance of the fixed effects in the GLMMs was calculated using the *z* distribution, and in the case of quasi-likelihood estimation correcting the standard errors of the coefficients as noted above. Visual inspection of diagnostic plots (qqplots and plots of the distribution of the residuals against fitted values) was checked to validate the models. We report mean ± standard error values throughout. Final sample sizes are indicated in Table A1, and Table A5 shows the means for the response variables for the different selection regimes across experiments. All the analyses were run in R 3.5.1 (R Core Team 2018).

Additional results

Extended results on effective population sizes (N_e)

Effective population sizes calculated using different methods (see above) ranged from around 41 to 51 across all selection regimes (Table A2). In the calculations of N_e in polygamous populations, implementing variation in the number of mating partners or accounting for fecundity between mating intervals has implications for effective population size, but the magnitude of the differences across scenarios is small (Table A3). Likewise, the effect of the observed rates of infertile matings on the estimated N_e is largely negligible (Table A4). It has to be noted that N_e estimations are not far from the number of breeding individuals. This is partly the result of three features of the study system and experimental procedures leading, in combination, to low variance in reproductive success (family size): i) infertility rates are extremely low in the system, with over 97% of singly mated females laying eggs (Rodriguez-Exposito and Garcia-Gonzalez, unpublished; Rodriguez-Exposito 2018), ii) two days for oviposition allow individual females to typically lay dozens of eggs (Zajitschek et al. 2018), and iii) random sampling of offspring in the selection experiment (and replicated in the calculations of N_e), ensuring even representation across families.

Baseline longevity

Neither the mating system, nor the spatial structure or their interaction had a significant effect on the baseline longevity of either sex (Table A6, and see Maklakov et al. 2007). Longevity was significantly determined by body size in both sexes (Table A6, Fig. A4), with larger individuals living for longer regardless of the selection regime under which they had evolved.

Extended results from the extreme levels of sexual conflict assay

We measured female longevity and LRS under conditions of extreme intensity of sexual interactions, where each female was exposed to continuous lifelong male harassment and mating attempts from three males. In tests of the evolution of male harm (effects of male

selection history on tester female fitness), there was a significant interaction between sexual selection and population subdivision selection histories on female LRS (see main text). In addition, female longevity was positively influenced by female body size (Table 4). The age of the female when entering the assay was also positively related to longevity, supporting the notion that females paid a reproductive cost when cohabiting with males (the older the female when entering the assay the longer she lived). Unlike the single mating situation, longevity and LRS were positively correlated. This is unsurprising as reversal of the longevity-offspring production trade-off is known to be context dependent in *C. maculatus* (Messina and Slade 1999; Messina and Fry 2003), and in this case, this reversal likely responds to the benefits that females obtain from multiple ejaculates (Savalli and Fox 1998; Zajitschek et al. 2018), including hydration benefits in this facultative aphagous capital breeder (Fox 1993; Arnqvist et al. 2005; Edvardsson 2007; Ursprung et al. 2009; Iglesias-Carrasco et al. 2018a). This survival-reproduction trade-off reversal, which is consistent across assays of evolution of male harm and female resistance under the conditions of male-biased sexual cohabitation, indicates that trade-offs between life-history traits in sexual conflict systems depends on a complex balance between the benefits and costs of sexual interactions.

The analysis of female resistance (effects of standardised males on focal female fitness) assessed after continuous sexual cohabitation showed a significant positive association between female age and LRS on longevity, and between female body size and longevity on LRS (Table 5). There were significant interactions between the experimental evolution treatments and female LRS on longevity (and between the mating system treatment and longevity on female LRS), and between metapopulation treatment and female body size on longevity (Table 5). We, however, refrain from drawing too many conclusions from these interactions, and we are in general cautious when interpreting the outcomes of our assays under conditions of continuous male-biased sexual cohabitation. Such continuous female exposure to male harassment and sexual interactions may impose extreme levels of sexual conflict that can be unrealistic in natural populations because of three reasons: extreme male-biased sex ratio, continuous lifelong sexual cohabitation, and highly reduced possibilities for females to escape male harassment (Zajitschek et al. 2018). The housing conditions in the continuous cohabitation assays, with three males per female in a confined space, may have actually compromised copulations because of the high levels of disruptions of mating attempts by competitor males under these conditions. It was not logistically possible to monitor mating rates in the continuous cohabitation tests and thus female mating frequency cannot be accounted for in the analyses. These assays may be therefore both less informative about the consequences of sexual interactions than the tests where mating frequency was taken into account (low and medium levels of sexual conflict assays).

Appendix Tables

Table A1.

Initial and final sample sizes relative to the number of individuals assayed in the analyses of longevity and lifetime reproductive success (LRS) across experiments. The missing data column indicates the number of individuals removed from the analyses due to sample deterioration or missing data. Data for females that did not produce any offspring due to absence of mating or LRS equal to zero were excluded (see text). Note that in the statistical analyses the unit of replication is the selection line (total number of replicated populations = 16).

Experiment/assays	Response	Generation	Initial N	Missing data	No offspring production	Final N
**No sexual conflict assay: Baseline longevity**						
Longevity	Female longevity	43	480	8	-	472
Longevity	Male longevity	43	480	8	-	472
**Low levels of sexual conflict assay (single mating test)**						
Male-induced harm	Female LRS	32	160	-	7	153
Male-induced harm	Female longevity	32	160	-	7	153
Female resistance	Female LRS	32	160	-	6	154
Female resistance	Female longevity	32	160	-	6	154
**Medium levels of sexual conflict assay (variable female mating rates)**						
Female resistance	Female longevity	12	112	0	-	112
Female resistance	Female longevity	30	160	10	-	150
**Extreme levels of sexual conflict assay (lifelong sexual cohabitation)**						
Male-induced harm	Female LRS	47	160	8	2	150
Male-induced harm	Female longevity	47	160	8	2	150
Female resistance	Female LRS	47	161	6	1	154
Female resistance	Female longevity	47	161	6	1	154

Table A2.

Average effective population sizes and CIs (in brackets) in the different selection regimes, calculated following simulations (see text). The first row of numbers within each selection regime (combination of metapopulation structure by mating system experimental evolution treatments) indicates the N_e estimate calculated summing the number of effective breeders. The second and third rows indicate N_e calculated using the number of effective breeders (always after accounting for variation in male reproductive success) to feed equation 1 or equation 3 (island model), respectively. The estimates for polygamous populations are for the case of 3 mates per female and correcting paternity after accounting for fecundity between mating intervals (for variations see Table A5).

	No metapopulation structure	Metapopulation structure
Polygamy	41.5 (37, 46)<br>41.3 (36, 46)<br>-	43.6 (39, 47)<br>43.4 (39, 47)<br>47.7 (39, 54)
Monogamy	45.9 (42, 50)<br>45.9 (42, 50)<br>-	46.6 (42, 50)<br>46.6 (42, 50)<br>50.7 (44, 54)

Table A3.

N_e variation in polygamous populations based on number of mating partners (2 or 3) per female, and on the correction for oviposition between matings (i.e., accounting for fecundity during mating intervals). Note that the more realistic situation is where a correction for fecundity between matings is simulated, as indicated in the text. The first row of numbers within each selection regime (combination of metapopulation structure by mating system experimental evolution treatments) indicates the N_e estimate calculated summing the number of effective breeders. The second and third rows indicate N_e calculated using the number of effective breeders (always after accounting for variation in male reproductive success) to feed equation 1 or equation 3 (island model), respectively. Average and 95% CIs (in brackets) N_e estimations are shown. *NSPoly*: Non-structure and polygamy; *SPoly*: Structure and polygamy. Average effective population sizes and CIs (in brackets) are given.

	Corrected N_e	Uncorrected N_e
NSPoly		
3 mates	41.5 (37, 46)<br>41.3 (36, 46)<br>-	39.4 (34, 44)<br>38.8 (34, 44)<br>-
2 mates	40.3 (36, 45)<br>39.9 (35, 45)<br>-	39.2 (34, 44)<br>38.6 (34, 44)<br>-
SPoly		
3 mates	43.6 (39, 47)<br>43.4 (39, 47)<br>47.7 (39, 54)	41.1 (36, 46)<br>40.7 (36, 46)<br>45.3 (34, 54)
2 mates	42.1 (37, 46)<br>41.8 (37, 46)<br>46.3 (34, 54)	40.9 (36, 45)<br>40.5 (35, 45)<br>45.0 (34, 54)

Table A4.

Effect of the observed rates of infertile matings on N_e. The estimates for polygamous populations are for the case of two mates per female (under higher female mating frequencies the reduction of N_e caused by infertility due to males is even more trivial). For simplicity only N_e estimates calculated summing the number of male and female effective breeders are shown. Likewise, the table only compares scenarios where a correction for oviposition between matings has been simulated (see files with the simulation structure and sampling protocol at https://doi.org/10.5061/dryad.r2280gbd9 for extended results). Average and 95% CIs (in brackets) N_e estimations are shown. *NSMono*: Non-structure and monogamy; *SMono*: Structure and monogamy; *NSPoly*: Non-structure and polygamy; *SPoly*: Structure and polygamy. Average effective population sizes and CIs (in brackets) are given.

	N_e	N_e - Infertile matings
NSMono	45.9 (42, 50)	44.5 (40, 48)
SMono	46.6 (42, 50)	45.1 (40, 48)
NSPoly	40.3 (36, 45)	40.2 (35, 45)
SPoly	42.1 (37, 46)	42.0 (37, 46)

Table A5.

Means, standard deviation (S.D.) and standard error (S.E) for the response variables (longevity, in days; LRS: number of adult offspring), for the different selection regimes across experiments. *NSMono*: Non-structure and monogamy; *SMono*: Structure and monogamy; *NSPoly*: Non-structure and polygamy; *SPoly*: Structure and polygamy. Note that females did not have access to oviposition substrate in the medium levels of sexual conflict assay and therefore longevity of females in this experiment is similar to longevity of virgin females (no sexual conflict assay) but it is sensibly higher than that in the other assays (low and extreme levels of sexual conflict) in which oviposition was allowed (Messina and Slade 1999; Messina and Fry 2003) (see text).

Experiment/assays	Response	Selection Regime	Mean	S.D.	S.E.	N
**No sexual conflict assay: Baseline longevity**						
Baseline longevity	Female longevity	*NSMono*	23.63	6.31	0.58	118
		SMono	23.08	5.03	0.46	120
		NSPoly	25.19	5.20	0.47	121
		SPoly	23.36	4.52	0.42	113
Baseline longevity	Male longevity	*NSMono*	16.88	3.93	0.36	117
		SMono	17.52	3.48	0.32	119
		NSPoly	18.58	3.41	0.31	118
		SPoly	17.62	3.28	0.30	118
**Low levels of sexual conflict assay (single mating test)**						
Male-induced harm	Female LRS	*NSMono*	54.15	13.60	2.18	39
		SMono	52.10	15.14	2.43	39
		NSPoly	55.06	13.19	2.20	36
		SPoly	56.41	11.74	1.88	39
Male-induced harm	Female longevity	*NSMono*	8.21	1.24	0.20	39
		SMono	8.26	1.04	0.17	39
		NSPoly	8.39	1.29	0.22	36
		SPoly	8.44	1.05	0.17	39
Female resistance	Female LRS	*NSMono*	66.78	17.57	2.89	37
		SMono	68.35	18.83	2.98	40
		NSPoly	63.77	14.18	2.27	39
		SPoly	71.05	13.58	2.20	38
Female resistance	Female longevity	*NSMono*	9.62	1.72	0.28	37
		SMono	9.73	1.34	0.21	40
		NSPoly	9.69	1.66	0.27	39
		SPoly	9.47	1.25	0.20	38
**Medium levels of sexual conflict assay (variable female mating rates)**						
Female resistance	Female longevity	*NSMono*	24.91	6.34	0.77	67
		SMono	27.95	5.33	0.69	60
		NSPoly	27.85	4.86	0.59	67
		SPoly	25.44	5.19	0.63	68
**Extreme levels of sexual conflict assay (lifelong sexual cohabitation)**						
Male-induced harm	Female LRS	*NSMono*	49.06	15.26	2.54	36
		SMono	49.97	20.35	3.35	37
		NSPoly	46.95	17.71	2.77	41
		SPoly	52.53	15.87	2.65	36
Male-induced harm	Female longevity	NSMono	7.39	1.29	0.22	36
		SMono	8.35	1.67	0.27	37
		NSPoly	8.51	1.70	0.27	41

		SPoly	7.44	1.32	0.22	36
Female resistance	Female LRS	NSMono	66.61	14.95	2.42	38
		SMono	69.11	15.64	2.54	38
		NSPoly	72.58	15.18	2.40	40
		SPoly	74.95	14.62	2.37	38
Female resistance	Female longevity	NSMono	7.37	1.15	0.19	38
		SMono	7.53	1.31	0.21	38
		NSPoly	7.43	0.98	0.16	40
		SPoly	8.00	0.81	0.13	38

Table A6.

Baseline Longevity. Linear mixed models (LMMs) on the effects of experimental evolution treatments on the longevity of virgin males and females. Monogamy is the reference level for the mating system treatment, and no population subdivision is the reference level for the metapopulation structure treatment. P-values were calculated using type II sums of squares on maximum likelihood models, while parameter estimates were calculated using REML models. P-values in bold are significant at <0.05.

**Fixed predictors**	*β*	*Type II Wald χ2*	*Wald test df*	*P-value*
**Females**				
Intercept	22.96			
Mating system treatment [Poly.]	0.63	0.03	1	0.868
Metapopulation structure treatment [Yes]	-0.56	0.04	1	0.851
Body size	9.93	328.73	1	**<<0.001**
Mating system * Metapop. structure	-1.19	0.01	1	0.928
Mating system * body size	8.33	1.60	1	0.206
Metapop. structure * body size	3.20	1.08	1	0.299
**Males**				
Intercept	18.02			
Mating system treatment [Poly.]	1.16	0.31	1	0.578
Metapopulation structure treatment [Yes]	0.59	0.01	1	0.917
Body size	12.83	35.74	1	**<<0.001**
Mating system * Metapop. structure	-1.05	0.53	1	0.465
Mating system * body size	2.35	0.27	1	0.603
Metapop. structure * body size	0.12	0.00	1	0.981

Appendix Figures

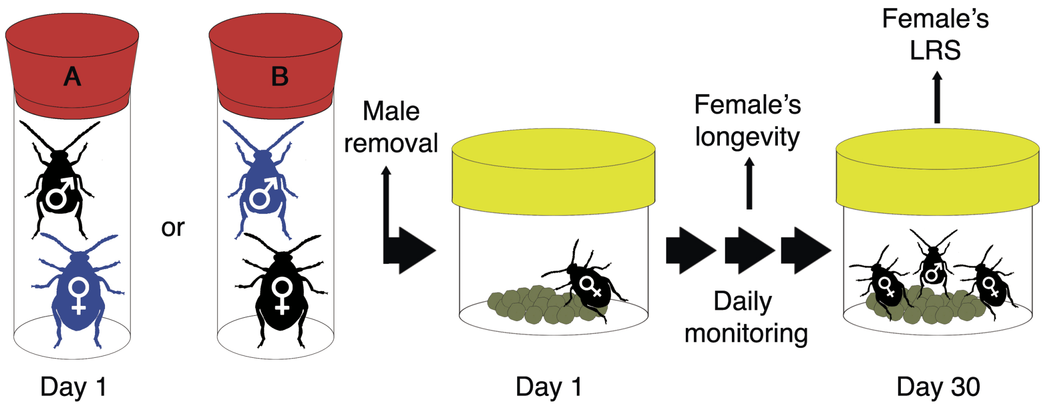

Figure A1.
Outline of the experimental design to measure male harm and female resistance after single mating. Each virgin focal individual (in black) was paired with a single tester individual (in blue) of the opposite sex. Afterwards, the male was removed and the female was transferred to a small plastic container (30 ml) with beans to allow oviposition. The oviposition container was checked daily to measure female longevity. Adult offspring emerged up to day 29th after the day the female mated provided a measure for LRS (see text).

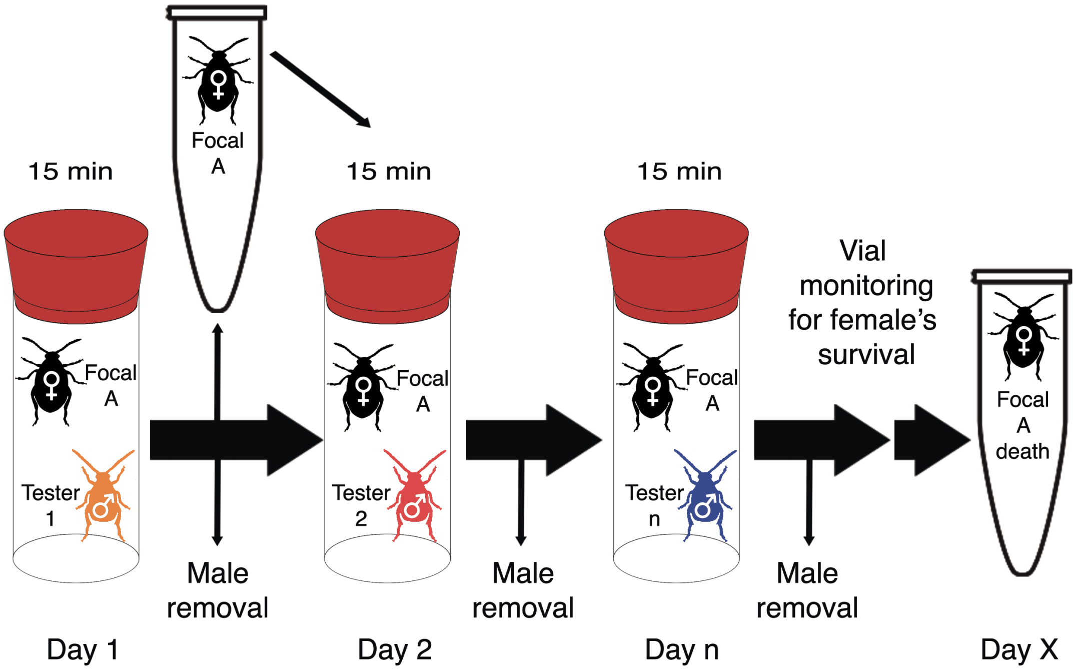

Figure A2.
Outline of the assays to measure female longevity when females were given multiple but controlled opportunities for mating/remating. Each female was placed daily (12 days for females from generation 12, 10 days for females from generation 30) with a standardized tester male (see text) in a glass vial for 15 minutes. At the end of that period the tester male was removed and the female was isolated in a 1.5 Eppendorf tube until the next mating opportunity 24 hours later. After all the mating opportunities were completed females remained in their isolation vials, which were checked once daily until female death. Day n* stands for the last day of the mating opportunities phase, and day x* stands for the day of female's death.

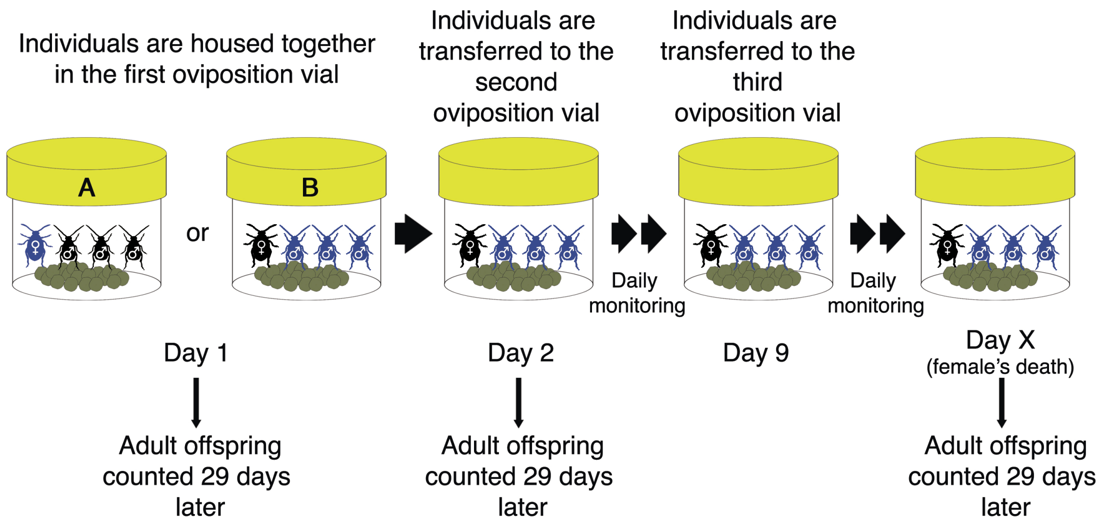

Figure A3.
Outline of the experiment to measure male harm and female resistance after lifelong cohabitation with individuals from the other sex. Male harm (A) was estimated in assays in which we measured longevity and LRS of tester females (in blue) housed with focal males (i.e., males from the selection lines; in black). Female's ability to resist the harm (B) was estimated in assays where longevity and LRS were assessed in focal females when they were housed with tester males (in blue). Each female (tester or focal) was housed with three males in a small plastic container (30 ml) with beans for the first 24 hours (day 1). On the second day, the individuals were transferred to a second oviposition container with new beans, where they remained for a week. Then, the individuals were transferred to a third oviposition container with new beans where they remained until female death. Adult offspring emerged up to day 29th after the day the experimental females were removed from the containers were counted, thereby providing a measure of LRS for each female (see text).

A

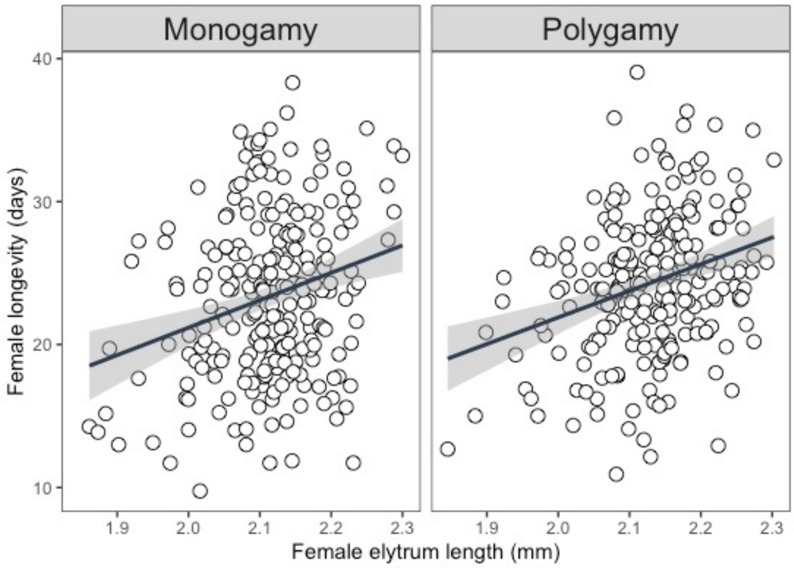

B

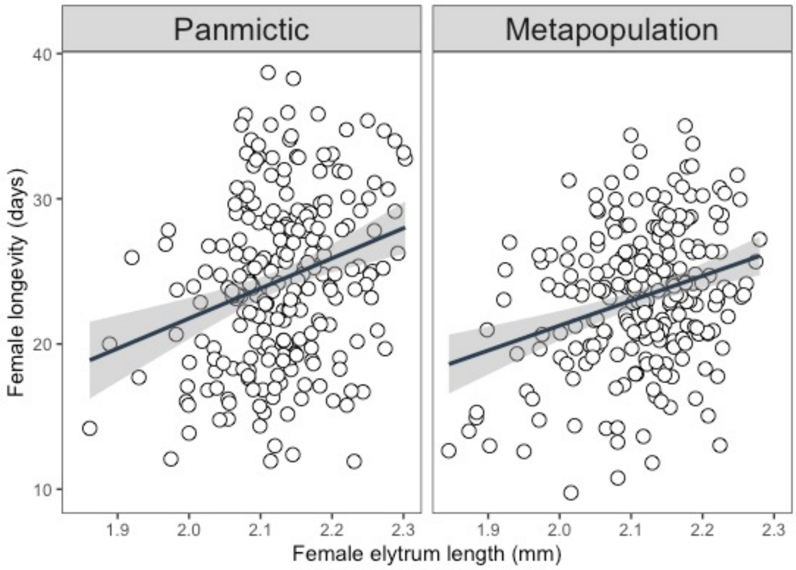

Figure A4.
Relationship between female body size and intrinsic female longevity (measured for virgin individuals) across females from selection regimes differing in selection associated with mating system (A), or differing in selection associated with metapopulation structure (B).

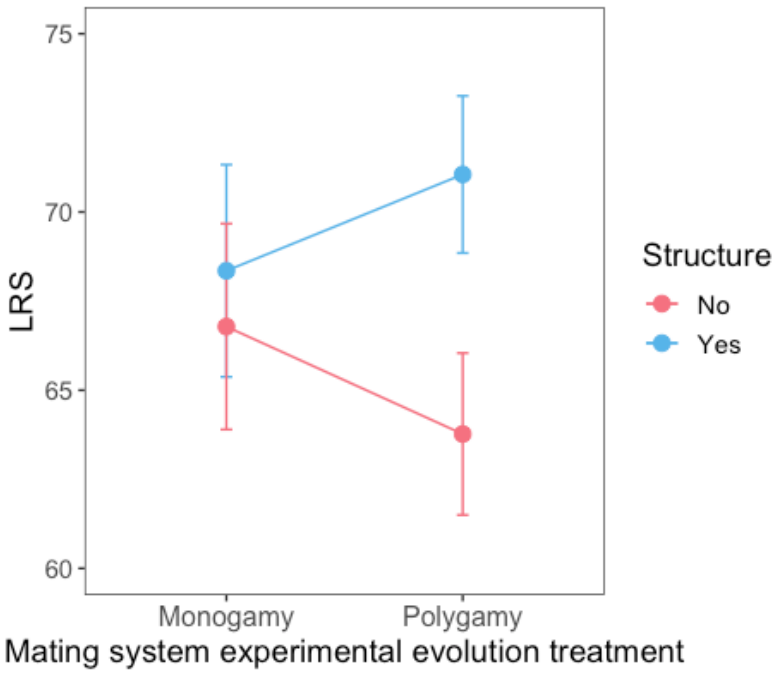

Figure A5.
Effects of mating system (monogamy vs. polygamy) and metapopulation structure (no vs. yes) in the evolution of focal females' LRS after single mating. The interaction is not significant and there is an effect of population spatial structure on LRS, with females from structured populations producing more adult offspring along their lifetime than females from non-structured populations (Table 2). Bars depict standard errors.

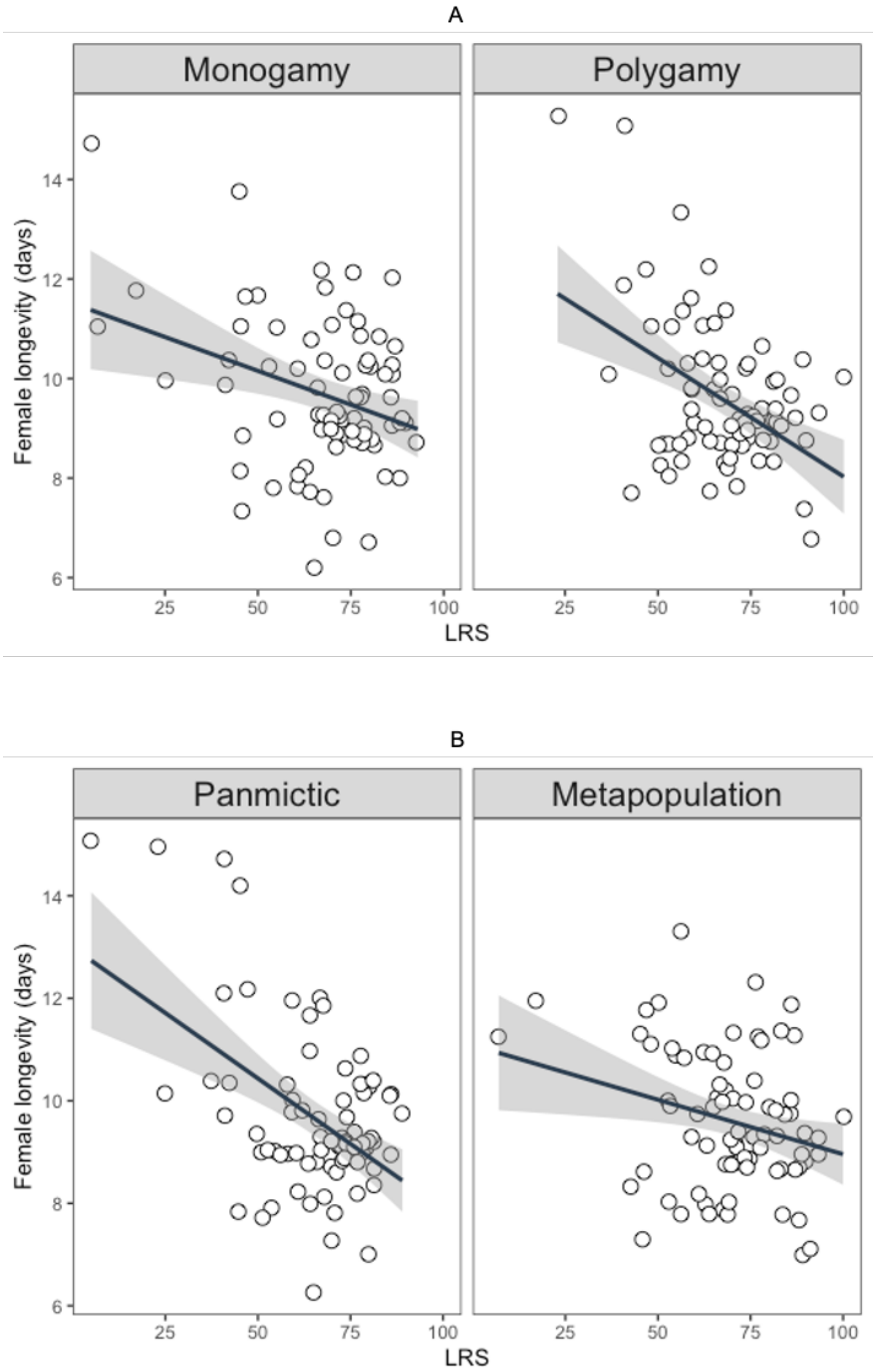

Figure A6.
Cost of reproduction: negative relationship between LRS and longevity across females from selection regimes differing in selection associated with mating system (A), or differing in selection associated with metapopulation structure (B), in assays in which females mated singly.

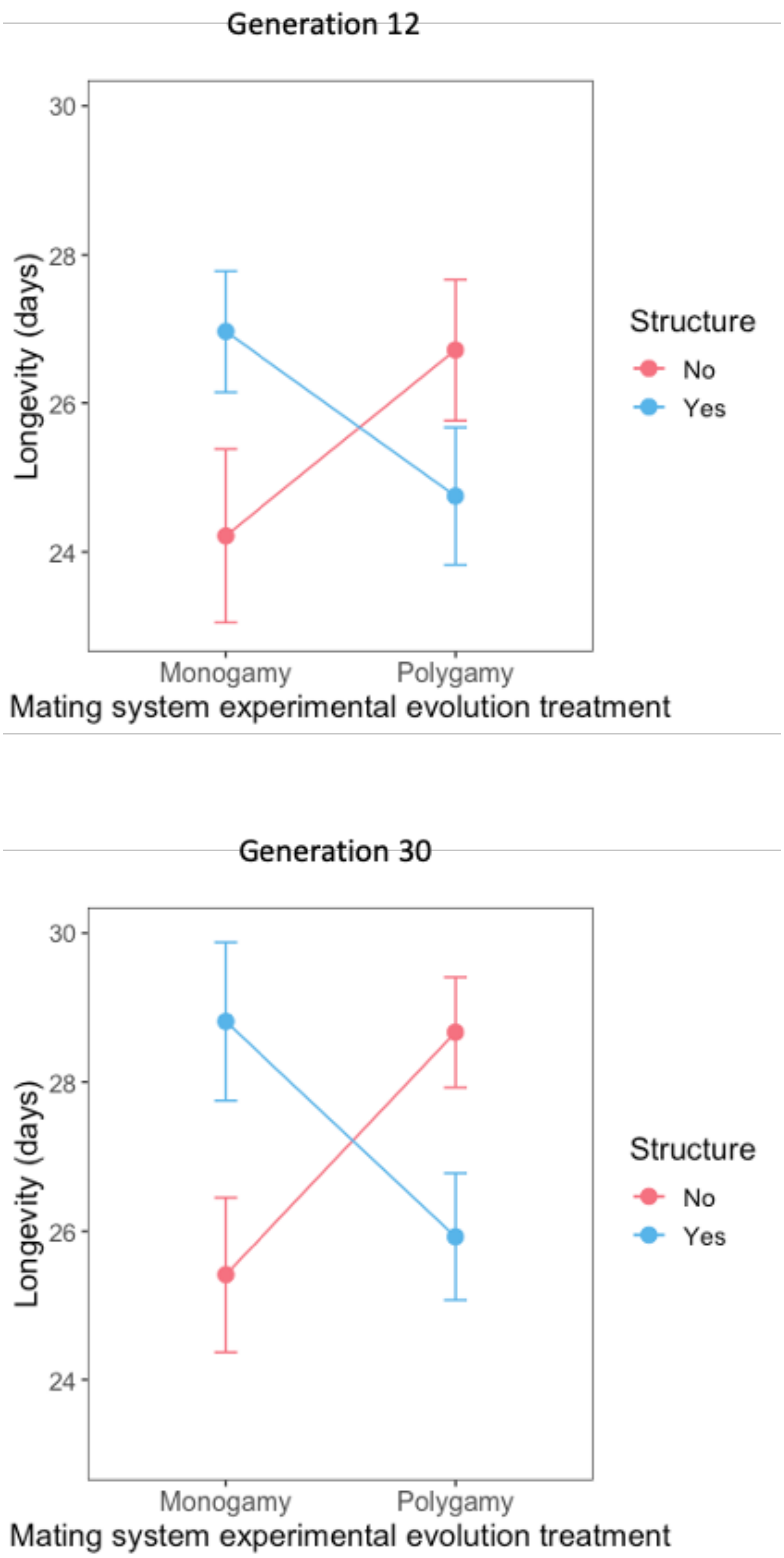

Figure A7.
Interaction of mating system (monogamy vs. polygamy) and metapopulation structure (no vs. yes) in the evolution of female resistance at generation 12 (left) and 30 (right) in assays with medium levels of sexual conflict (see text).

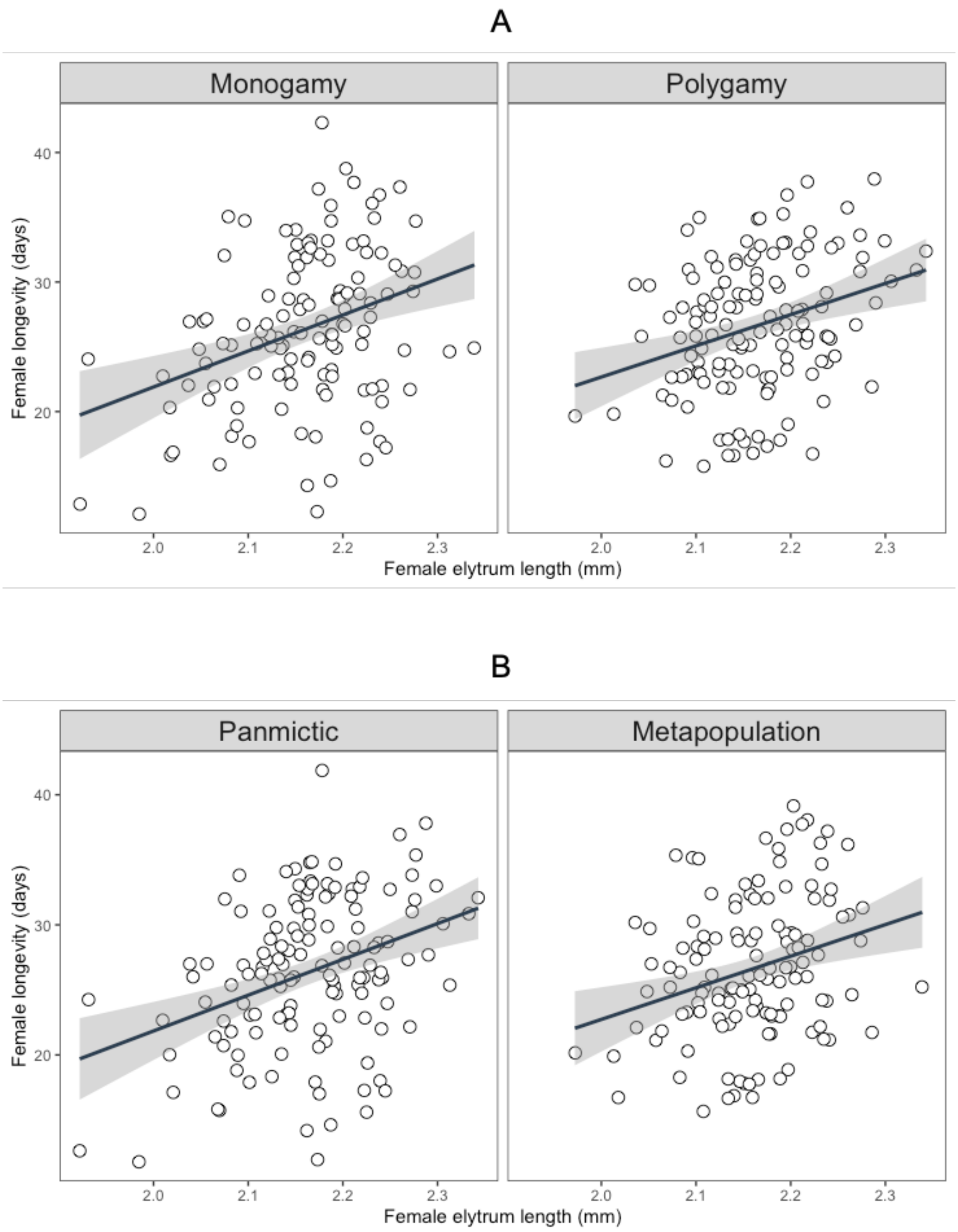

Figure A8.
Relationship between female body size and female longevity across females from selection regimes differing in selection associated with mating system (A) or differing in selection associated with metapopulation structure (B) in assays with medium levels of sexual conflict.

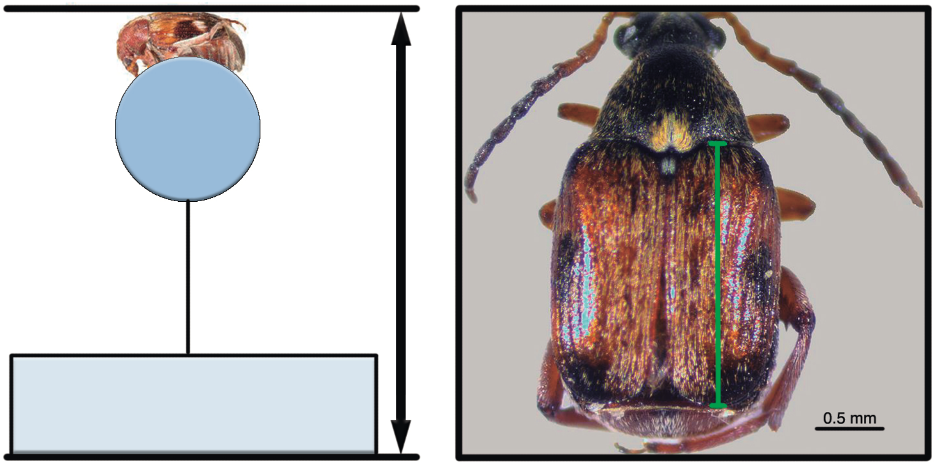

Figure A9.
Measurement of elytron length. The image shows the placement of the specimen on a fixed pedestal, and the dorsal view of an individual. Elytron length (in millimeters) was always measured on the right elytron. The measurement was always carried out parallel to the edge and connecting the most distant points of the right wing.

References

Arnqvist, G. 2020. Mixed models offer no freedom from degrees of freedom. TREE 35:329-335.

Arnqvist, G., T. Nilsson, and M. Katvala. 2005. Mating rate and fitness in female bean weevils. Behav. Ecol. 16:123-127.

Barr, D. J., R. Levy, C. Scheepers, and H. J. Tily. 2013. Random effects structure for confirmatory hypothesis testing: Keep it maximal. J. Mem. Lang. 68:255-278.

Bates, D., M. Mächler, B. Bolker, and S. Walker. 2015. Fitting linear mixed-effects models using lme4. J. Stat. Softw. 67:1-48.

Becker, W. A. 1984. *Manual of quantitative genetics*. Pullman, Washington.

Berg, E. C. and A. A. Maklakov. 2012. Sexes suffer from suboptimal lifespan because of genetic conflict in a seed beetle. Proc. R. Soc. Lond. B 279:4296-4302.

Berger, D., K. Grieshop, M. I. Lind, J. Goenaga, A. A. Maklakov, and G. Arnqvist. 2014. Intralocus sexual conflict and environmental stress. Evolution 68:2184-2196.

Berger, D., I. Martinossi-Allibert, K. Grieshop, M. I. Lind, A. A. Maklakov, and G. Arnqvist. 2016. Intralocus sexual conflict and the tragedy of the commons in seed beetles. Am. Nat. 188:E98-E112.

Bilde, T., U. Friberg, A. A. Maklakov, J. D. Fry, and G. Arnqvist. 2008. The genetic architecture of fitness in a seed beetle: assessing the potential for indirect genetic benefits of female choice. BMC Evol. Biol. 8:295.

Canal, D., F. Garcia-Gonzalez, and L. Z. Garamszegi. 2021. Experimentally constrained early reproduction shapes life history trajectories and behaviour. Sci. Rep. 11:4442.

Cayetano, L., A. A. Maklakov, R. C. Brooks, and R. Bonduriansky. 2011. Evolution of male and female genitalia following release from sexual selection. Evolution 65:2171-2183.

Crow, J. F. and M. Kimura. 1970. *An introduction to population genetics theory*. Harper and Row, New York.

Crudgington, H. S. and M. T. Siva-Jothy. 2000. Genital damage, kicking and early death. Nature 407:855-856.

Czesak, M. E. and C. W. Fox. 2003. Genetic variation in male effects on female reproduction and the genetic covariance between the sexes. Evolution 57:1359-1366.

Dougherty, L. R. and L. W. Simmons. 2017. X-ray micro-CT scanning reveals temporal separation of male harm and female kicking during traumatic mating in seed beetles. Proc. R. Soc. Lond. B 284:20170550.

Eady, P. 1994. Intraspecific variation in sperm precedence in the bruchid beetle *Callosobruchus maculatus*. Ecol. Entomol. 19:11-16.

Eady, P. and S. Tubman. 1996. Last-male sperm precedence does not break down when females mate with three males. Ecol. Entomol. 21:303-304.

Eady, P. E. 1991. Sperm competition in *Callosobruchus maculatus* (Coleoptera: Bruchidae): a comparison of two methods used to estimate paternity. Ecol. Entomol. 16:45-53.

Eady, P. E., P. Rugman-Jones, and D. V. Brown. 2004. Prior oviposition, female receptivity and last-male sperm precedence in the cosmopolitan pest *Callosobruchus maculatus* (Coleoptera: Bruchidae). Anim. Behav. 67:559-565.

Edvardsson, M. 2007. Female *Callosobruchus maculatus* mate when they are thirsty: resource-rich ejaculates as mating effort in a beetle. Anim. Behav. 74:183-188.

Falconer, D. S. and T. F. C. Mackay. 1996. *Introduction to Quantitative Genetics*. Longman, Essex.

Fox, C. W. 1993. Multiple mating, lifetime fecundity and female mortality of the bruchid beetle, *Callosobruchus maculatus* (Coleoptera: Bruchidae). Funct. Ecol. 7:203-208.

Fox, C. W., M. L. Bush, D. A. Roff, and W. G. Wallin. 2004. Evolutionary genetics of lifespan and mortality rates in two populations of the seed beetle, *Callosobruchus maculatus*. Heredity 92:170-181.

Garcia-Gonzalez, F. 2004. Infertile matings and sperm competition: the effect of "Nonsperm Representation" on intraspecific variation in sperm precedence patterns. Am. Nat. 164:457-472.

Garcia-Gonzalez, F. and D. Dowling, K. 2015. Transgenerational effects of sexual interactions and sexual conflict: non-sires boost the fecundity of females in the following generation. Biol. Lett. 11:20150067.

Gay, L., D. J. Hosken, P. Eady, R. Vasudev, and T. Tregenza. 2010. The evolution of harm—Effect of sexual conflicts and population size. Evolution 65:725-737.

Hood, G. M. 2008. *PopTools version 3.0.6. Available on the internet. URL http://www.cse.csiro.au/poptools*.

Hotzy, C. and G. Arnqvist. 2009. Sperm competition favors harmful males in seed beetles. Curr. Biol. 19:404-407.

Hotzy, C., M. Polak, Johanna L. Rönn, and G. Arnqvist. 2012. Phenotypic engineering unveils the function of genital morphology. Curr. Biol. 22:2258-2261.

Iglesias-Carrasco, M., G. Bilgin, M. D. Jennions, and M. L. Head. 2018a. The fitness cost to females of exposure to males does not depend on water availability in seed beetles. Anim. Behav. 142:77-84.

Iglesias-Carrasco, M., M. D. Jennions, S. R. K. Zajitschek, and M. L. Head. 2018b. Are females in good condition better able to cope with costly males? Behav. Ecol. 29:876-884.

Maklakov, A. A., C. Fricke, and G. Arnqvist. 2007. Sexual selection affects lifespan and aging in the seed beetle. Aging Cell 6:739-744.

McNamara, K. B., S. P. Robinson, M. E. Rosa, N. S. Sloan, E. van Lieshout, and L. W. Simmons. 2016. Male-biased sex ratio does not promote increased sperm competitiveness in the seed beetle, *Callosobruchus maculatus*. Sci. Rep. 6:28153.

McNamara, K. B., N. S. Sloan, S. E. Kershaw, E. van Lieshout, and L. W. Simmons. 2020. Males evolve to be more harmful under increased sexual conflict intensity in a seed beetle. Behav. Ecol. 31:591-597.

Messina, F. J. and J. D. Fry. 2003. Environment-dependent reversal of a life history trade-off in the seed beetle *Callosobruchus maculatus*. J. Evol. Biol. 16:501-509.

Messina, F. J. and A. F. Slade. 1999. Expression of a life-history trade-off in a seed beetle depends on environmental context. Physiol. Entomol. 24:358-363.

Pitnick, S. and F. Garcia-Gonzalez. 2002. Harm to females increases with male body size in *Drosophila melanogaster*. Proc. R. Soc. Lond. B 269:1821-1828.

R Core Team. 2018. *R: A language and environment for statistical computing*. R Foundation for Statistical Computing, Vienna, Austria. http://www.R-project.org.

Rice, W. R. and B. Holland. 2005. Experimentally enforced monogamy: Inadvertent selection, inbreeding, or evidence for sexually antagonistic coevolution? Evolution 59:682-685.

Rodriguez-Exposito, E. 2018. Evolutionary responses to the independent and interacting action of sexual selection and population spatial structure: Insights from experimental evolution in a species with sexual conflict. PhD Thesis, Universidad de La Laguna.

Savalli, U. M. and C. W. Fox. 1998. Genetic variation in paternal investment in a seed beetle. Anim. Behav. 56:953-961.

Sayadi, A., A. Martinez Barrio, E. Immonen, J. Dainat, D. Berger, C. Tellgren-Roth, B. Nystedt, and G. Arnqvist. 2019. The genomic footprint of sexual conflict. Nat. Ecol. Evol. 3:1725–1730.

Schielzeth, H. 2010. Simple means to improve the interpretability of regression coefficients. Methods Ecol. Evol. 1:103-113.

Schielzeth, H. and W. Forstmeier. 2009. Conclusions beyond support: overconfident estimates in mixed models. Behav. Ecol. 20:416-420.

Simmons, L. W. 2001. *Sperm Competition and its Evolutionary Consequences in the Insects*. Princeton University Press, Princeton.

Snook, R. S., L. Brüstle, and J. Slate. 2009. A test and review of the role of effective population size on experimental sexual selection patterns. Evolution 63:1923-1933.

Ursprung, C., M. den Hollander, and D. T. Gwynne. 2009. Female seed beetles, *Callosobruchus maculatus*, remate for male-supplied water rather than ejaculate nutrition. Behav. Ecol. Sociobiol. 63:781-788.

Vasudeva, R., D. C. Deeming, and P. E. Eady. 2014. Developmental temperature affects the expression of ejaculatory traits and the outcome of sperm competition in *Callosobruchus maculatus*. J. Evol. Biol. 27:1811-1818.

Walsh, J. B. and M. Lynch. 2018. *Evolution and Selection of Quantitative Traits*. Oxford University Press, Oxford, UK.

Wright, S. 1951. The genetical structure of populations. Ann. Eugen. 15:323–354.

Yasui, Y. and F. Garcia-Gonzalez. 2016. Bet-hedging as a mechanism for the evolution of polyandry, revisited. Evolution 70:385-397.

Zajitschek, S. R. K., D. K. Dowling, M. L. Head, E. Rodriguez-Exposito, and F. Garcia-Gonzalez. 2018. Transgenerational effects of maternal sexual interactions in seed beetles. Heredity 121:282-291.

Zuk, M., F. Garcia-Gonzalez, M. E. Herberstein, and L. W. Simmons. 2014. Model systems, taxonomic bias, and sexual selection: Beyond *Drosophila*. Ann. Rev. Entomol. 59:321-338.

Zuur, A., E. N. Ieno, N. Walker, A. A. Saveliev, and G. M. Smith. 2009. *Mixed Effects Models and Extensions in Ecology with R*. Springer, New York.
